# Unlocking nature’s treasure-chest: screening for oleaginous algae

**DOI:** 10.1038/srep09844

**Published:** 2015-07-23

**Authors:** Stephen P. Slocombe, QianYi Zhang, Michael Ross, Avril Anderson, Naomi J. Thomas, Ángela Lapresa, Cecilia Rad-Menéndez, Christine N. Campbell, Kenneth D. Black, Michele S. Stanley, John G. Day

**Affiliations:** 1 Microbial and Molecular Biology Department, Scottish Association for Marine Science (SAMS), Scottish Marine Institute, Oban, PA37 1QA, UK; 2 Culture Collection of Algae and Protozoa (CCAP), Scottish Association for Marine Science (SAMS), Scottish Marine Institute, Oban, PA37 1QA, UK; 3 Ecology Department, Scottish Association for Marine Science (SAMS), Scottish Marine Institute, Oban, PA37 1QA, UK

## Abstract

Micro-algae synthesize high levels of lipids, carbohydrates and proteins photoautotrophically, thus attracting considerable interest for the biotechnological production of fuels, environmental remediation, functional foods and nutraceuticals. Currently, only a few micro-algae species are grown commercially at large-scale, primarily for “health-foods” and pigments. For a range of potential products (fuel to pharma), high lipid productivity strains are required to mitigate the economic costs of mass culture. Here we present a screen concentrating on marine micro-algal strains, which if suitable for scale-up would minimise competition with agriculture for water. Mass-Spectrophotometric analysis (MS) of nitrogen (N) and carbon (C) was subsequently validated by measurement of total fatty acids (TFA) by Gas-Chromatography (GC). This identified a rapid and accurate screening strategy based on elemental analysis. The screen identified *Nannochloropsis oceanica* CCAP 849/10 and a marine isolate of *Chlorella vulgaris* CCAP 211/21A as the best lipid producers. Analysis of C, N, protein, carbohydrate and Fatty Acid (FA) composition identified a suite of strains for further biotechnological applications e.g. *Dunaliella polymorpha* CCAP 19/14, significantly the most productive for carbohydrates, and *Cyclotella cryptica* CCAP 1070/2, with utility for EPA production and N-assimilation.

Many micro-algal taxa accumulate lipid to high levels, usually in the form of non-polar glycerolipids such as triacylglycerol (TAG) and often including DAG, MAG (di-, mono-)^1^,^2^,^3^,^4^,^5^. Generally the increase in total lipid in algal cells occurs on entering stationary phase or with nutrient depletion or other stresses^2^. In general this consists primarily of the neutral lipids, such that TAGs can account for up to 80% of the total cellular lipids^2^. Consequently membrane lipids (polar lipids such as phospholipids and galactolipids) comprise a minor fraction in these cases^2^. This capacity to produce non-polar lipid combined with an ability to photosynthesise and generate biomass efficiently has stimulated considerable interest in growing algae at large scale as a feedstock for biofuels and other biotechnological products^1^,^2^,^3^,^4^,^5^. Moreover, many micro-algae can thrive in seawater/brackish conditions, or higher salinity, temperature, or under extreme pH^1^,^6^. These capabilities could reduce dependence on freshwater supplies of future large-scale production facilities and thus minimise competition with traditional agriculture for resources. Such algae are also likely to cope with increases in ionic strength due to evaporative losses^7^. A supply of N and phosphates are still required from fertilizers, or by wastewater input for nutrient supply^8^. In both cases strains that can assimilate available N efficiently are desirable.

Large scale production is a requisite for biomass/biofuel production, where prime considerations include productivity, ease of cultivation, harvesting and non-polar lipid extraction^6^,^9^,^10^,^11^. Other important considerations include the Fatty Acid (FA) composition of extractable lipids with specific regard to the desired final use of the biomass product. Micro-algal omega-3 long chain PUFA’s such as Eicosapentanoic acid (EPA or 20:5n-3) and Docosahexaenoic acid (DHA or 22:6n-3) can be transferred up the food chain, thereby adding health benefits and commercial value to feeds and human dietary supplements^12^,^13^,^14^. These two FA command the highest premium in the latter case, but there is evidence that their precursors such as Stearidonic (SDA or 18:4n-3) are also beneficial^13^. Western diets are known to have insufficient EPA and DHA, but there is also evidence that the overall omega-3 to omega-6 long-chain (C_≥18_) PUFA ratio in dietary FA intake is important (in the United States this dietary intake ratio is estimated to be ~1:10 but the optimum is thought to be 1:6)^13^,^15^. Consequently the levels of certain low value omega-3 PUFA’s such as α-linolenic acid (ALA) are also significant^13^,^16^. Paradoxically, one omega-6 PUFA, γ-linolenic acid (GLA or 18:3n-6), is known to have anti-inflammatory roles and to be beneficial in cardiovascular disease^16^. Quantification of TFA through direct derivatization and GC-FID is an appropriate approach to assessing potential as dietary omega-3 long-chain PUFA can be absorbed effectively, irrespective of whether they are present in non-polar lipids or in membrane lipids (e.g. phospholipids in Krill biomass)^17^. Furthermore, certain biodiesel production processes convert TFA directly by transesterification on biomass^18^.

In terms of biofuels, polyunsaturated FA’s (PUFA’s) are considered less desirable in lipids destined for biodiesel, due to issues of oxidative stability^19^,^20^. Conversely, cold-flow issues relating to high saturate levels can also be problematic especially limiting their use in specialist areas such as jet fuels^19^,^20^,^21^. Biofuels rich in short-chain C_10-14_ methyl esters (ME) (e.g. Coconut) have been successfully used as a component of aviation fuels^22^. It is reasonable to expect that screening a diverse range of micro-algal phyla for useful and unusual FA compositions could pay dividends^14^.

There are currently relatively few micro-algae grown commercially, i.e. profitably, at large-scale, with the most widely cultivated “alga” the cyanobacterium *Spirulina* having low levels of non-polar lipid^8^. The few taxa making up the bulk of the algal biomass market are mostly extremophiles (*Spirulina*, *Dunaliella*), or freshwater (*Chlorella*)^8^. There is a paucity of commercially viable strains for growing in seawater, yet the most conservative estimates suggest that at least 72,500 algae exist (projected figures for diatoms alone range from 20,000 up to 2 million species)^23^. Approximately 44,000 species have been described and 73% of these are currently documented in AlgaeBase; many cross-referenced to collections such as the Culture Collection of Algae and Protozoa (CCAP) at SAMS^23^,^24^. Studies have shown that strain-to-strain variation in yields are often highly significant, thus it is sensible to test, not only exemplar taxa, but also multiple strains from specific species^1^. Even without isolating new strains this is a huge undertaking therefore development of rapid and reliable methods to identify the most promising strains for further analysis is required. Elemental analysis offers several advantages: robust data, small sample size and nil sample preparation, but has yet to be exploited in high-throughput screening of micro-algae.

The value of isolating new strains from saline water bodies was recognized by the US DoE Aquatic Species Program (ASP) which ran for two decades and ended in 1996^7^. Earlier screens such as these utilized rapid, semi-quantitative dye-staining method for primary screening for non-polar lipids. But this method has limitations when making comparisons between strains and can underestimate levels in some organisms with significant biotechnological potential such as the *Chlorella*^7^,^25^. Since the ASP close-out report there has been no published large-scale screens of micro-algal collections for non-polar lipid (oil) production^7^. A recent screen of the SAG collection focussed only on FA composition and produced no data on total lipid content or biomass production^26^. With increased interest in value-added co-products in biofuel production, there is also a need to consider high-value fatty acids and products other than lipids such as carbohydrates and protein^12^.

The principal aim of this study was to identify a suite of model strains for large scale/low cost biotech purposes such as biodiesel, biogas and added-value nutraceutical feeds. The focus was on strains capable of good growth at seawater salinity, with somewhat low nitrate and phosphate levels and no CO_2_ supplementation. This reflected the high economic cost of providing fertilizer and CO_2_ at large scale. Growth conditions were standardized in order to set a baseline with which to make a comparative analysis of the strains. A further goal was to investigate the partitioning of resources in algae; such a comparative dataset could also be linked to omic and phylogenetic data-sets in the future. A final aim was to improve methodological strategies and facilitate larger-scale screening.

## Results

### Screening for growth in artificial seawater

**Figure 1:**
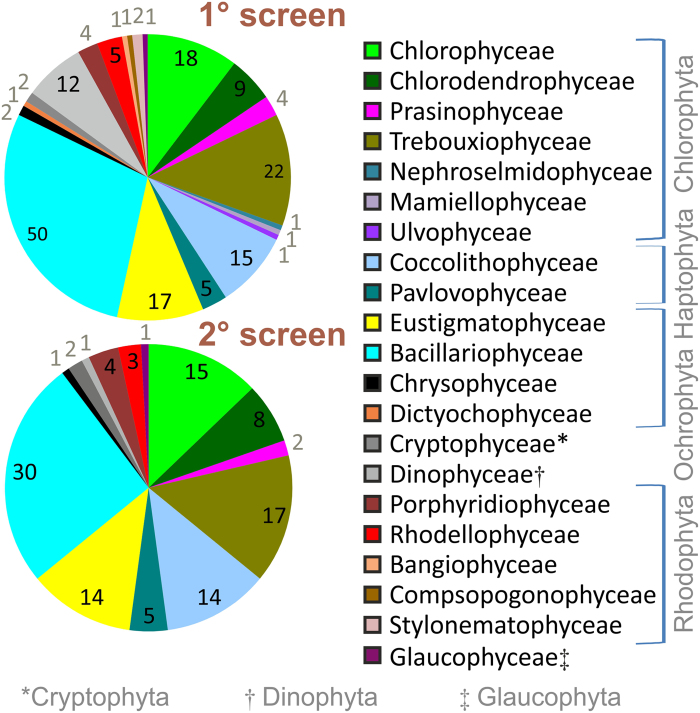
Taxonomic distribution of micro-algal strains. The 175 strains subjected to the primary screen for growth under standard conditions and the 117 selected for the secondary screen for composition and yield measurements are indicated by pi-charts. Colour-coding designates taxa to 21 protistan classes covering 7 phyla. Number of strains per class is indicated on the pi-chart. Data tabulated in Supplementary Dataset S1-3 online.

**Figure 2:**
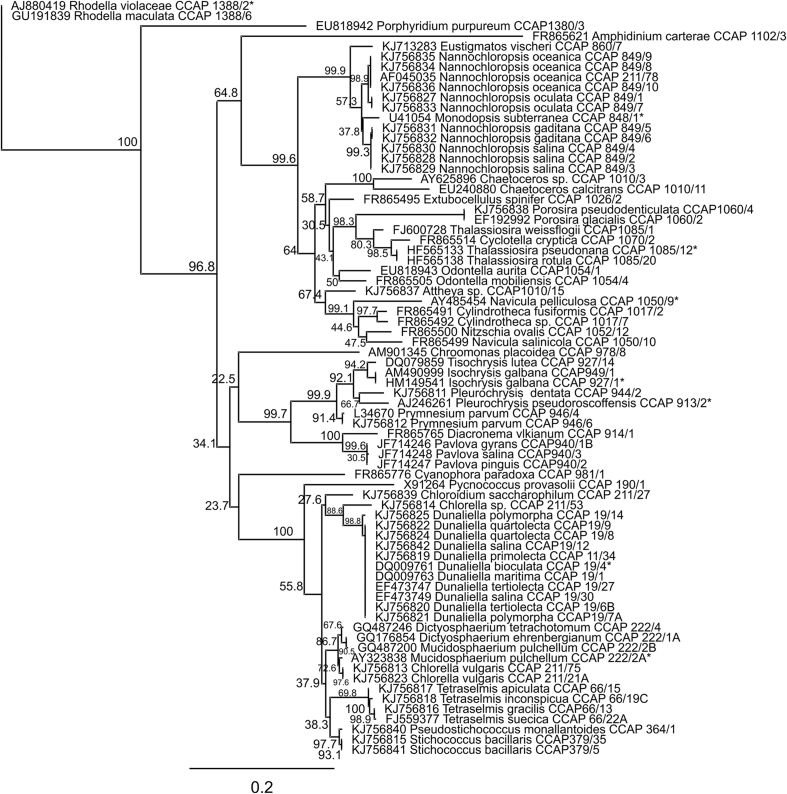
Molecular phylogeny of the screened algae. This was inferred from a comparison of 18S rDNA sequences from the micro-algal strains studied. The resultant tree was generated from a maximum likelihood analysis with Bootstrap percentage values indicated where N = 1000. Strains are labelled according to 18S GenBank accession; name and CCAP culture collection accession. Where denoted (*) the sequence was derived from the same strain held in other collections.

The CCAP collection maintains approximately 3000 protistan and cyanobacterial strains of which about 600 are marine micro-algae^24^. A total of 175 strains were short-listed for screening, based on their stability in long-term culture (Supplementary Dataset S1 online). Approximately 50% were isolated in the UK/UK territorial waters and the rest were of diverse origins world-wide (Supplementary Dataset S1 online). The taxonomic affiliations of the short-listed strains and a graphical summary of the outcome of the primary screen are depicted in Fig. 1 and phylogenetic origins depicted in Fig. 2. In the primary screen, strains were tested for growth in defined media and 33% were rejected due to poor growth leaving 117 that entered the secondary screen (Supplementary Dataset S2 online). The majority of strains studied were salt-tolerant and 103 grew well in artificial seawater-based medium (f/2), with relatively low nitrate (1 mM) under standard conditions (see methods) (Supplementary Dataset S3 online). A subset of 11 strains (*Haematococcus* and *Dictyosphaerium*), were grown in freshwater medium with similar nitrate levels (JM) and a minority, 3 strains, were grown in high nitrate freshwater medium (3NBBM+V) (*Monodopsis subterraneae* CCAP 848/1, *Eustigmatos vischeri* CCAP 860/7 and *Desmodesmus elegans* CCAP 258/8) (Supplementary Dataset S3 online). Red algal strains (Rhodophyceae and Porphyridiaceae) and Dinophyceae were grown under lower light according to reported requirements (see methods)^27^. Analysis of biomass yield and composition was carried out in the secondary screen (Supplementary Dataset S4-7 online).

### High biomass strains of diverse phylogenetic origin

Biomass quantified by dry-weight (DW) or combustive MS elemental analysis of C is tabulated (Supplementary Dataset S4 online). A close correlation was observed between biomass yields (gl^−1^) or productivities (gl^−1^d^−1^) measured by the two methods (Pearson’s coefficient 0.828 and 0.877, P < 0.001; Supplementary Fig. S1 online) although C content as a function of DW ranged widely from 7-66% (mean 40%, RSD 28%). Overall, C was chosen as a better measure of valuable biomass.

**Figure 3:**
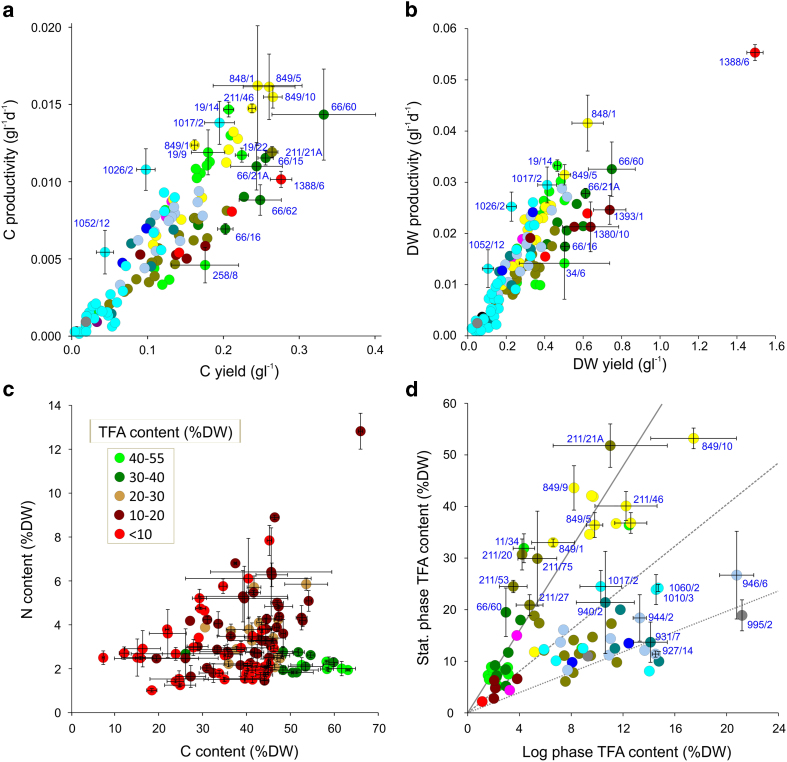
Biomass and TFA production levels of the algae screened. Biomass productivity and yields are shown in terms of (**a**) C content and (**b**) DW. (**c**) Analysis of N and C content depicted in relation to TFA content, which is indicated by colour coding. (**d**) Stationary and log phase TFA content in DW biomass (ratios 4:1, 2:1 and 1:1 indicated by diagonals). Data points in (**a**), (**b**), (**d**) are colour coded by class as defined in Fig. 1 (2° screen) and error bars (SD) are depicted for key algae labelled according to CCAP strain accession no. Data are derived from replicate batch cultures (tabulated in Supplementary Datasets S4-5 online).

The distribution of biomass yields and productivities for the screen are also shown graphically by mass C and DW (Fig. 3a,b). Ranked data are also shown for those strains exceeding the 70^th^ percentile for both C yield and C productivity (Table 1). The first 8-9 strains ranked by these two parameters were not significantly different (t-test P > 0.05) and there were four strains in common: *M. subterranea* CCAP 848/1; *Nannochloropsis gaditana* CCAP 849/5; *Nannochloropsis oceanica* CCAP 849/10 and *Tetraselmis* sp. CCAP 66/60. *Rhodella violaceae* CCAP 1388/6 had significantly highest DW yield and productivity (t-test, P = 0.002 and P = 0.014) (Fig. 3b), but had low C content biomass (18% DW); hence C yield ranked 2^nd^ and C productivity 21^st^ (Table 1).

A close correlation between C yield and C productivity data was evident (Pearson’s coefficient 0.887, P < 0.001), although the relationship was significantly influenced by taxonomic grouping; either by class or genus (MANOVA, P < 0.001) (Fig. 3a). Comparing species and strains from two of the high yielding genera: *Nannochloropsis* group mean overall growth rates were found to be significantly higher than for *Tetraselmis* (0.060 c.f. 0.042 d^−1^; t-test P < 0.001), although mean C yields (respectively 0.191 and 0.229 gl^−1^) were not significantly different (t-test P = 0.138) (Supplementary Dataset S4 online). The small cell-size of the Eustigmatophytes investigated here (<3 μm diameter), compared with *Tetraselmis* sp. (>20 μm) could be a contributory factor in accounting for the higher productivity observed^10^,^28^. Nevertheless, among the diatoms screened, *Cylindrotheca fusiformis* CCAP 1017/2 was the most productive and it was characterized by very large pennate cells (length 100 μm: www.ccap.ac.uk) (Table 1). Ranked within the top eight strains for productivity it was not significantly different from *N. oceanica* CCAP 849/10 (P = 0.14), but had lower yields than this strain (P = 0.006, Table 1). The diatoms *Extubocellulus spinifer* CCAP 1026/2, *Chaetoceros simplex* CCAP 1085/3 and *Cyclotella cryptica* CCAP 1070/2 exceeded the 70^th^ percentile for C productivity, but were below this percentile for C yield (Supplementary Dataset S4 online). In addition to *Tetraselmis*, among the green algae, several *Dunaliella* strains and *Chlorella vulgaris* CCAP 211/21A were productive (Table 1). Of the haptophyte species, *Isochrysis* sp. CCAP 927/12 and *Pleurochrysis pseudoroscoffensis* CCAP 961/3 were also productive, but the remainder clustered around biomass yield and productivity means (Table 1, Fig. 3a).

### High TFA content strains identified by MS analysis

A three-way comparison of N and C content determined by MS, along with TFA content determined by GC-FID (Fig. 3c). TFA content was an indication of total useful lipids present (non-polar and membrane glycerolipids). This analysis defined 94% of high TFA content strains (>30% TFA per DW) according to their C-content ≥48%DW and N-content <3%DW. The 16 species/strains thus selected were from the genera: *Nannochloropsis*, *Chlorella* and *Dunaliella*, whereas the outlier was *Haematococcus pluvialis* CCAP 34/6. The relationship between TFA content and C/N parameters was confirmed as significant as follows. Grouping by TFA content as defined in Fig. 3c, MANOVA gave P < 0.001 for C/N parameters and one-way ANOVA for N-content and C-content, gave P = 0.003 and P < 0.001. Post-hoc analysis using Fisher’s comparison indicated significant difference in relation to C-content (P < 0.05) where TFA was >40% DW c.f. <40% and >30% DW c.f. <20%; in relation to N-content, >30% DW c.f. 10-20% and 10-20% c.f. <10% was likewise significant.

Within the group of 16 high-TFA strains defined above by high C and low N, protein ranged from 5-15% DW (mean 8.8%) and carbohydrate ranged from 9-26% DW (mean 14%) (Supplementary Dataset S4 online). The mean levels of this group were significantly less (t test, P < 0.001) than those strains also having ≥48%DW C, but with higher N, >3%DW N (6 strains); here protein ranged from 14-16% DW (mean 14.7%) and carbohydrate ranged from 14-44% (mean 31%). Therefore high C content can indicate glycerolipids or other hydrocarbons (75-85% C by mass)^29^ but high levels of protein (53% C, 16% N)^30^, or other organic amines might also be responsible. This latter scenario would be revealed by high N content; indicating less C-partitioning into lipids. Conversely, partitioning towards carbohydrates (44% C)^30^ would tend to reduce overall C content. In practice, >90% of the strains with high TFA levels (>30% DW) could be identified solely by MS analysis for N and C content. This provides the means for formulating a rapid and robust strategy for future screening for high TFA content.

### Defining high TFA-producer strains

The full data-set for TFA content (%DW), TFA yield and TFA productivity are tabulated (Supplementary Dataset S4 online). Strains exceeding the 70^th^ percentile for the three parameters were ranked (Table 2). There was no correlative support for a trade-off between biomass (C) productivity and TFA content (Supplementary Fig. S2 online). TFA content in the screen ranged from 1-53% (mean 16%, RSD 71%), the highest being *N. oceanica* CCAP 849/10 (53% DW) and *C. vulgaris* CCAP 211/21A (52% DW). That of the *N. oceanica* strain was significantly higher than all other isolates tested (t-test, P = 0.025) except for *C. vulgaris* CCAP 211/21A (P = 0.63) and *H. pluvialis* CCAP 34/6 (36% DW, P = 0.054) (Table 2). TFA productivity by *N. oceanica* CCAP 849/10 was the highest in the screen (P = 0.040) except for *N. gaditana* CCAP 849/5 (NS, P = 0.14) which had lower TFA content: 36% DW (P < 0.001). TFA productivity of *C. vulgaris* CCAP 211/21A was somewhat lower than *N. oceanica* CCAP 849/10 (by 19%; P = 0.013) and that of *H. pluvialis* CCAP 34/6 was substantially less than the *Nannochloropsis* strain (by 66%; P < 0.001). In terms of TFA yield, top ranking strains were *C. vulgaris* CCAP 211/21A followed by *N. oceanica* CCAP 849/10 (Table 2). Therefore *N. oceanica* CCAP 849/10 and *C. vulgaris* CCAP 211/21A emerged as the only strains combining high TFA content (>50% DW) with high TFA yields and TFA productivity.

*C. vulgaris* CCAP 211/21A appeared to be exceptional in these terms among the marine *Chlorella*-like strains tested (i.e. *Chlorella* and *Chloroidium* sp.) (t-tests, P = 0.001-0.02) (Supplementary Dataset S4 online). Although *N. oceanica* CCAP 849/10 was the best *Nannochloropsis* strain tested, all 12 species/strains examined were in the high TFA-producing subset (Table 2). Comparison of 18S rRNA and ITS genomic DNA sequences defined 5 of the strains as *N. oceanica* species (Fig. 2; Supplementary Fig. S3 online). These were distinguished by higher mean TFA contents (>40%DW compared with the rest of the species/strains in the genus, which ranged 33-37%DW: t-test on group means, P = 0.002). They also had on average 45% higher yields and productivity (group means t-test within genus, P = 0.035-0.046) (Table 2). In terms of estimated evolutionary distance, *N. oceanica* strains were closer to *N. oculata* than *N. salina* or *N. gaditiana* (Supplementary Fig. S3 online). Despite this relatedness, *N. oculata* strains had the lesser TFA productivities within this genus (Table 2). Protein content was also 60% higher in the latter compared with *N. oceanica* (10.3% c.f. 6.4%; t-tests comparing the individual strains: P = 0.001-0.02), suggesting species-specific differences in C-partitioning (Supplementary Dataset S4 online).

Concerning the other promising species listed (Table 2), *H. pluvialis* CCAP 34/6, because of its complex life-cycle and relatively slow growth, has limited potential for commercial lipid production^31^. A single *Dunaliella* strain, *D. primolecta* CCAP 11/34, out of 11 tested was identified as having similarly high TFA content and productivity (P > 0.05), but yield was half that of *H. pluvialis* (P = 0.046). Five diatom species, including the four identified above for highest biomass productivity, had moderately high TFA contents (20-25%; differences NS.) (Table 2). Here, *C. fusiformis* CCAP 1017/2, also the best diatom for biomass, ranked the highest for TFA content, yield and productivity (significant for TFA yield and productivity c.f. *Cyclotella cryptica* CCAP 1070/2: P = 0.019-0.027).

Comparison of stationary phase and log phase TFA content (Fig. 3d, Supplementary Dataset S5 online) indicated that a group mainly comprising the highest lipid accumulators (*Nannochloropsis* species, marine *Chlorellas* and high lipid *Dunaliella*) had on average 4 times more TFA at stationary phase than in log phase. The most productive of these strains would seem best suited to a fed-batch cultivation mode. A second group, comprising the haptophytes, cryptophytes, diatoms and freshwater Trebouxiophyceans tended to accumulate at least half of the TFA during log phase. The most productive lipid accumulators in this category (for instance *C. fusiformis* CCAP 1017/2) might also be suitable for semi-continuous production methods.

### Sequestration of supplied N in biomass

Screen data obtained for N and protein content, yields, productivities, C/N ratio and N ratio are tabulated (Supplementary Dataset S4 online). Protein levels measured by Lowry assay correlated closely with N content as determined by elemental analysis (Pearson’s correlation coefficient = 0.812, P < 0.001; Supplementary Fig. S4 online) giving a mean N ratio of 3.66 (RSD = 21%). An indirect correlation was noted between N content and either C yield or C productivity (Pearson’s = 0.641 and 0.493, P < 0.001). A similar relationship was also noted between protein content and these C parameters (Pearson’s = 0.536 and 0.394, P < 0.001) (Supplementary Fig. S5-6 online). Despite this potential caveat, a core set of about 7-10 strains above the 70^th^ percentile for content, yield and productivity in terms of protein or N were identified (Table 3). Although these included the small subset of 3 strains in the screen that were cultivated in high nitrate freshwater medium (3NBBM+V): *M. subterranea* CCAP 848/1, *E. vischeri* CCAP 860/7 and *D. elegans* CCAP 258/8, the rest were grown in standard saline f/2 medium. In terms of N-productivity, the former strain was at least 2-fold higher than any other micro-alga in the screen (t-test, P = 0.015). Although this strain also ranked highest for protein productivity and yield, it was not significantly higher in this respect than *Tisochrysis lutea* CCAP 927/14 or *Chlorella vulgaris* CCAP 211/75, both of which were grown on standard f/2 (P > 0.05, Table 3).

**Figure 4:**
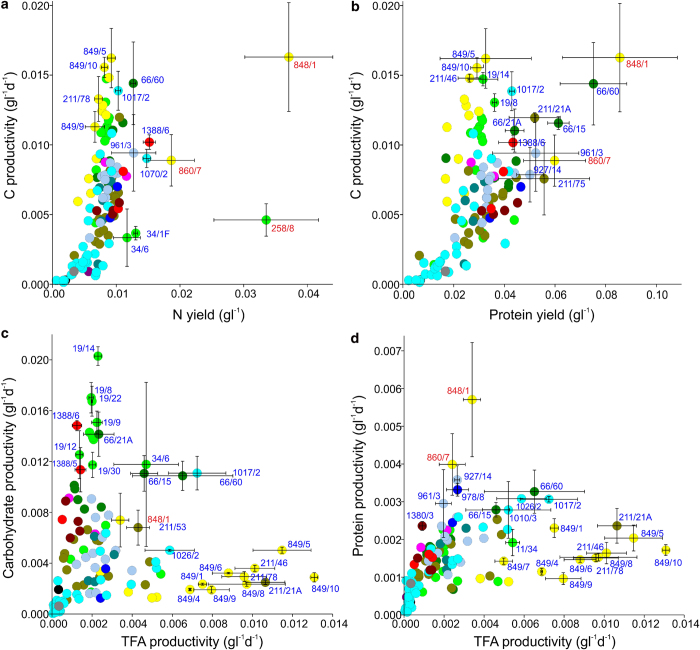
C and N resource partitioning in the algal screen. C productivities are depicted in comparison with assimilation of supplied N in terms of (**a**) N culture yield (**b**) protein. Comparison of TFA production levels with (**c**) carbohydrate and (**d**) protein. Data points are colour coded by class as defined in Fig. 1 (2° screen) and error bars (SD) are depicted for key algae labelled according to CCAP strain accession number (red text indicates strains grown in 3NBBM+V). Data are from 117 strains and are derived from replicate batch cultures (tabulated in Supplementary Dataset S4 online).

The amount of N that was assimilated into biomass from the medium was indicated by N culture yield (and to an extent by protein yield), and these data are compared graphically with biomass C productivity (Fig. 4a,b). The amount of N supplied in the standard low nitrate saline f/2 media (a majority of those studied i.e. 103 strains) was 0.0124 gl^−1^; this was similar in the low nitrate freshwater JM medium (utilised to cultivate 11 of the strains studied) at 0.0156 gl^−1^ and for high nitrate freshwater 3NBBM+V medium (utilised for 3 strains) this was 0.1236 gl^−1^. The mean N yield for strains in f/2 was 0.0076 gl^−1^, but with the higher C productivity strains, N yields tended to approach the amount of N supplied (Fig. 4a). Here, *R. violaceae* CCAP 1388/6, *C. cryptica* CCAP 1070/2 and *Tetraselmis* sp. CCAP 66/60 retained more N than the *Nannochloropsis* species studied (Fig. 4a). In contrast the 3 strains in high nitrate (3NBBM+V) assimilated <30% of supplied N (Fig. 4a). In two of these, *M. subterranea* CCAP 848/1 and *Desmodesmus elegans* CCAP 258/8, this equated to significantly more N accumulated than the rest of the screen (t-test, P = 0.015 and P = 0.045; Fig. 4a; Table 3), but was only associated with high protein yields in the former strain (Fig. 4b). The C/N ratio at stationary phase harvest correlated with C productivity and yields for the strains grown in relatively low nitrate media (Supplementary Fig. S7 online). Of the best producing strains, for those growing in f/2 the C/N ratio was in the region of 30-40 and for *M. subterannea* CCAP 848/1 in 3NBBM+V, this was 8.4.

### Carbohydrate synthesis and C partitioning

Carbohydrate levels assessed using Dubois ranged from 3-81% DW (mean 30% and RSD 58%) (Supplementary Dataset S4 online). A similarly wide spread of data about the mean was noted for FA, but was less evident for protein or N (above). A three-way comparison of TFA, carbohydrate and protein productivities is shown graphically (Fig. 4c,d) and these data are also ranked for the top producing strains in Supplementary Fig. S8 online. Hence a great degree of flux control variation in C-partitioning between TFA and carbohydrate was apparent with most of the high biomass (C) producers focussing either on carbohydrate or TFA. A few of the high C producers (e.g. *Tetraselmis* sp. CCAP 66/60, *C. fusiformis* CCAP 1017/2), grown on standard low nitrate f/2, achieved a balance between TFA, carbohydrate and protein production (Fig. 4c,d; Supplementary Fig. S8 online).

*Dunaliella, Tetraselmis, Rhodella* and *Haematococcus* species were the most productive for carbohydrate and *Dunaliella* polymorpha CCAP 19/14 emerged as the most productive strain (c.f. rest of screened P = 0.016, except *H. pluvialis* CCAP 34/6: P = 0.0856) (Fig. 4c, Table 3). *R. violaceae* CCAP 1388/6 was the highest yielding strain studied (P = 0.018 c.f. rest of screen, except *H. pluvialis* CCAP 34/6: P = 0.9) (Supplementary Dataset S4 online). Particularly high carbohydrate content was observed in both *H. pluvialis* strains: CCAP 34/6 and CCAP 34/1F at 68-81% DW; the former significantly higher than other strains (P = 0.026, except *H. pluvialis* CCAP 34/1F P > 0.05 and *Mucidosphaerium pulchellum* CCAP 222/2B P = 0.065). All the *Dictyosphaerium*/*Mucidosphaerium* species tested showed high carbohydrate (Supplementary Dataset S4 online), and as previously observed, mucilaginous exudate was apparent in these strains^32^.

### Analysis of FA composition and micro-algal phylogeny

**Figure 5:**
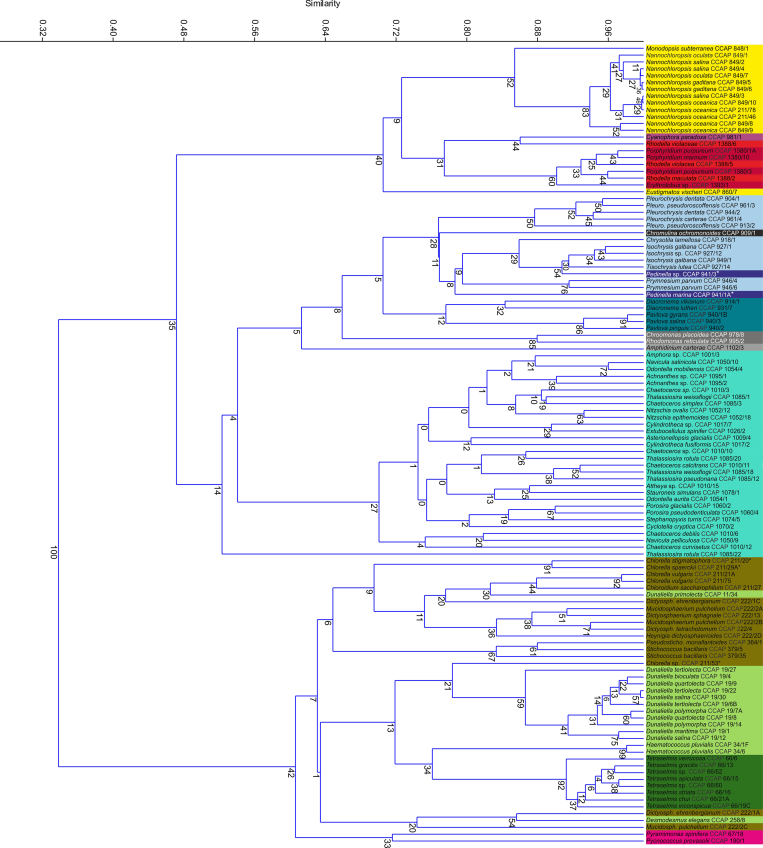
Cluster analysis of FA compositional data. A data cut-off of 0.1% was applied and data (mol%) clustered using a PAST algorithm employing Rho parameters (bootstrap value N = 1000). Micro-algal classes were defined by the colour-coding scheme in Fig. 1 (2° screen). Strains undergoing taxonomic review are indicated (*); see Supplementary Text S1 online. Data tabulated in Supplementary Dataset S6 online.

A cluster analysis of FA compositional data for the screen is shown (Fig. 5, data in Supplementary Dataset S6 and Fig. S9 online). Hierarchical cluster analysis of the FA data separated the green algae from the chromistan and red algae (Fig. 5). Here distinct patterns of C_16_ desaturation have been attributed to the action of distinct plastidial desaturases substrate specificities in the red and green algal lineages^33^. Clustering of FA compositional data led to further grouping of most strains by phyla, class and in some cases according to genus. The outcome of this exercise was most successful with the Prymnesiophytes, where FA composition appeared to vary along taxonomic lines. Conversely, in diatoms there appeared to be substantial compositional variation at the species and even the infra-species level. Further analysis of FA composition and phylogenetics is presented in Supplementary Text S1 online.

### Implications for biofuels

The best biofuel strains identified were *N. oceanica* CCAP 849/10 and *C. vulgaris* CCAP 211/21A based on content and productivity (Table 2). PUFA levels can negatively impact biodiesel storage in proportion to their unsaturation^19^,^20^. In the screen as a whole, PUFA levels were high compared with current biofuel feedstocks, with a mean of 34%, but ranged from 4-74%, (Supplementary Dataset S6 online)^19^,^20^. These were relatively high at 32% in *C. vulgaris* CCAP 211/21A, but confined to tri-unsaturates or less (Supplementary Dataset S6 online). All the *Nannochloropsis* strains had low PUFAS’s (5-11% TFA) of which about half was EPA. The high levels of 16:0 and 16:1n-7 in *N. oceanica* CCAP 849/10 led to a mean chain length among the lowest in the screen at 16.4 (Supplementary Dataset S6 online). This would be expected to be an improvement over palm oil for instance, where 16:0 and 18:1n-9 are the dominant FA, and where cold flow issues exist^19^,^20^. Several diatoms and haptophytes, had C_14_ saturate levels ranging from 20-40% TFA (highest *Odontella mobiliensis* CCAP 1054/4), but accompanied by high amounts of C_20-22_ PUFA’s. This would diminish cold flow problems (albeit with oxidation issues from the latter), but high C_14_ was not observed among the most productive TFA strains (Supplementary Datasets S4, S6 online). Nevertheless, model strains for genetic engineering or breeding could be found: *C. fusiformis* CCAP 1017/2, the best diatom TFA/biomass producer, was 7% C_14_, but other species/strains in the genus had up to 30%, e.g. *Cylindrotheca* sp. CCAP 1017/7. In the haptophytes, C_14_ was at 20-28% in *Prymnesium parvum* CCAP 946/4, *Pavlova salina* CCAP 940/3 and *Isochrysis* sp. CCAP 927/12, but with moderate total TFA contents and productivities (Supplementary Dataset S6 online).

### High-value FA producing strains

FA composition was analysed further in productivity terms for the commercially significant FA such as EPA, DHA, SDA and GLA (Supplementary Dataset S7 online, Table 4). The two best strains for EPA production were *M. subterranea* CCAP 848/1 and *C. cryptica* CCAP 1070/2 (Table 4). The *M. subterranea* strain studied (also held in the UTEX algal collection as UTEX 151) is a known EPA source strain and was used as benchmark although it is normally grown in N-rich freshwater media similar to that used here^34^,^35^. Other *C. cryptica* strains have been used in mariculture^36^,^37^. The former Eustigmatophycean strain ranked highest in EPA yield and productivity, but this was not significantly more than *C. cryptica* CCAP 1070/2 (t-test P = 0.09 for both parameters) (Table 4). Although *M. subterranea* CCAP 848/1 (and fellow Eustigmatophycean *E. vischeri* CCAP 860/7) had higher EPA FA composition (22% cf. 16%; t-test to *C. cryptica* CCAP 1070/2: P < 0.001 and P = 0.007), total TFA content per biomass was only 8-12% cf. 20% DW (P < 0.05) and with less EPA per biomass (2-3%DW cf. 4%; P < 0.05, Table 4). Therefore, under the screen conditions used here, the *C. cryptica* CCAP 1070/2 strain appeared to be a more promising source of EPA and possibly a suitable mariculture strain due to the higher EPA biomass content and capability for productive growth on low-N saline f/2 medium.

With respect to DHA, the most productive strains were confined to the haptophytes, where *T. lutea* CCAP 927/14 ranked the highest (see Table 4 for significance). This was combined with high DHA FA content (16%) which was only exceeded by the much less productive dinoflagellate *A. carterae* CCAP 1102/3 (19%, P = 0.002) and *Pedinella marina* 941/1A (18% but NS, P = 0.68); otherwise it was significantly higher than rest of screen (P = 0.023). The *T. lutea* CCAP 927/14 strain is extensively used in aquaculture^38^ because of its suitable nutritional profile. However, the generally low TFA content of the examined Isochrysidales order, of 11-16% DW, would not favour them for non-polar lipid extraction. Significantly higher DHA per biomass was observed in *Prymnesium parvum* CCAP 946/4 and CCAP 946/6 (Table 4), with relatively high TFA content at 16-26% DW. DHA content at 13-14% TFA was slightly less than *T. lutea* CCAP 927/14 (P = 0.003 and 0.023) although DHA productivity was not significantly less than that of *T. lutea* CCAP 927/14 (P = 0.60 and 0.12; comparing all parameters between *P. parvum* strains: NS, P > 0.05) (Table 4). Given that members of this genus produce a suite of toxins against fish and protozoa^39^, commercial use may be limited, if resolvable, through genetic means. Fish oil based feeds and dietary supplements often have similar levels of both EPA and DHA. In this regard, three Pavlovophyceaen strains were productive for both FA, of these *Diacronema lutheri* CCAP 931/7 was the most productive for DHA and is extensively used in aquaculture^40^. However, another related species, *Pavlova salina* CCAP 940/3, showed more balanced EPA and DHA levels, combined with TFA content of 20% DW (Table 4).

Haptophytes were also high producers of SDA, a precursor of EPA and DHA^14^(Supplementary Table S1 online); the most productive being *T. lutea* CCAP 927/14 (significantly higher c.f. rest of screen, P = 0.040). Its SDA FA composition was highest in the screen (17%, P = 0.008), excepting *A. carterae* CCAP 1102/3 (32%, P < 0.001). SDA productivity was also high in *Chroomonas placoidea* CCAP 978/8 and *Pleurochrysis dentata* CCAP 944/2, with the latter having favourable TFA content at just under 20% DW.

Regarding GLA composition, the freshwater alga *D. ehrenbergianum* CCAP 222/1A at 6% TFA was comparable to the principal commercial source, Evening Primrose oil (5-10%); significantly higher c.f. rest of screen (P = 0.009)(Supplementary Table S1 online)^41^. This strain was the most productive for GLA along with *H. pluvialis* CCAP 34/6; the diatoms *C. muelleri* CCAP 1010/3 and *C. fusiformis* CCAP 1017/2 (NS difference, P > 0.05).

It was also instructive to examine the complete complement of omega-3 long chain PUFA in the screen (Supplementary Table S2 and Dataset S6-7 online). Although the health benefits, and commercial premiums, of individual omega-3 long chain PUFA are known to differ, a high ω-3/ω-6 ratio is thought to be beneficial in dietary fat. The mean ratio (≥C_18_ PUFA) for the screen was high at 8.4, compared with western intake (~0.1) but varied greatly from 0.1-74 (Supplementary Dataset S6 online). The lowest ratios were due to high levels of Linoleic acid (LA or 18:2n-6) or Arachidonic acid (ARA or 20:4n-6), or both (e.g. *Porphyridium*). Mean omega-3 long-chain PUFA content in TFA in the screen was also high at 23% (RSD 53%) and ranged from 2-68%. The highest was *Amphidinium carterae* CCAP 1102/3 (t-test P < 0.001), due to SDA, DHA and EPA (Supplementary Table S2 and Dataset S6 online). However, this strain was not productive under the conditions employed and produces toxins^42^. When taking growth into account, a group of 11 strains lay above the following 70^th^ percentiles: omega-3 long-chain PUFA composition (i.e. ≥28%), content in biomass, yield and productivity (Supplementary Table S2 online). Most of these strains had TFA contents below 20% DW however. In fact a weak inverse-relationship was present between omega-3 long-chain PUFA (and total PUFA) composition in relation to TFA content in biomass (Supplementary Fig. S10 online). But interestingly, the high TFA content (55% DW) strain *C. vulgaris* CCAP 211/21A, had significantly the highest omega-3 long-chain PUFA productivity in the screen (t-test P = 0.013) (Supplementary Fig. S11 and Table S2 online). Although this strain lacked potential commercially high-premium FA, the FA composition appeared to be beneficial from a dietary perspective with relatively high ALA (13%), oleic acid (48%) and low LA (10%); ω-3/ω-6 ratio 1.2 (Supplementary Dataset S6 online). By comparison, most major plant seed oil extracts such as Canola/rapeseed or sunflower tend to have low omega-3 long-chain PUFA content, with LA a major if not the predominant unsaturated FA^20^.

## Discussion

The aim of this work was to screen a micro-algal collection for strains of biotechnological potential. The focus was primarily on marine strains and the key objective was to identify high lipid producers, with additional measurements to provide a complete compositional analysis. This screen was carried out at medium-scale with 0.4 L culture volumes to yield sufficient biomass for several assays. However, it was found that elemental analysis for C and N content alone was sufficient to identify strains with high TFA content (>30%DW). This procedure requires only 1 mg DW (from as little as 2 mL culture) encapsulated in foil with a run-time of 8 min per sample. Therefore, for future screening, this procedure would allow a faster processing time and a significant scale-down of culture volume leading to higher throughput (more so than is possible with GC of directly trans-esterified FA^43^ or FTIR spectroscopy^44^). Only the Nile-Red plate assay approach would be faster, but this technique has limitations in accuracy relating to between-species comparison, dye uptake and carotenoid interference^25^.

The two highest lipid producers were *Nannochloropsis oceanica* CCAP 849/10 and a marine *Chlorella vulgaris* CCAP 211/21A strain. The former was originally isolated from a fish hatchery (Table 5, Supplementary Dataset S1 online) and, since many freshwater *Chlorella* are already commercially exploited, it is likely that both strains would be robust enough for use in open-air ponds^8^,^45^. This *Chlorella* is the first salt-tolerant strain with noted potential and the TFA content observed (52% DW) is similar to some of the higher reported levels in the literature for its freshwater relatives (48-57% DW; gravimetric measurements of total lipid)^46^. Given the high levels of ALA and oleic acid relative to LA, the lipid composition of this strain represents a dietary improvement over mainstay vegetable oils, which are usually high in LA^20^. On the basis of current commercial *Chlorella* production levels and the potential to increase these, opportunities exist for products in niche health-food markets, but in future a greater impact on dietary quality might be possible^13^,^15^,^20^. The FA composition of lipids from *Nannochloropsis* species, along with sunflower and Canola, are more suited for biodiesel production^19^,^20^. A detailed analysis of 12 different *Nannochloropsis* strains from 4 species found that *N. oceanica* strains had significantly higher TFA productivity and content than the others tested. This suggested that here phylogenetic origin was the major factor involved, rather than the local origin and/or the associated adaptations of the different strains to their local environments. Observing a significant relationship between phylogenetic and biochemical data at the species level indicated that the methods used in the screen were robust. It was noted that the aforementioned *Chlorella* and *Nannochloropsis* high lipid-producing strains accumulated most product in stationary phase. In contrast, haptophytes and diatom strains, where the most productive strain was *C. fusiformis* CCAP 1017/2 (Table 5), showed much less temporal variation in TFA accumulation. It was apparent that different phylogenetic groups should be grown using different cultivation methods, based on these data.

A subset of 20 strains is listed in Table 5 that was found to be the most productive for the specific storage products: lipid, carbohydrate and protein, and algal biomass. Interestingly, 3 of the top 8 biomass producers were isolated from commercial aquaculture sites, although the majority of strains entering the screen were originally collected from the natural environment (Table 5). It is likely that such strains will have undergone artificial selection predisposing them to mass culture^10^. In addition to flagging up previously unstudied strains, 4 out of 20 the highlighted strains from the screen were of previously known potential (Table 5): *N. oceanica* CCAP 211/46 and 211/78, *M. subterranea* CCAP 848/1 and *T. lutea* CCAP 927/14^35^,^38^,^44^,^47^. This also demonstrated the robust nature of the methods employed in screening. The most promising source of total carbohydrate was *D. polymorpha* CCAP 19/14 (Table 5).

It was also notable that the top strains emerged from several different taxonomic phyla with varying latitudes of origin, from sub-tropical (e.g. *T. lutea* CCAP 927/14 from Hawaii) to cool temperate (*R. violaceae* CCAP 1388/6 from Sweden). Several had originally been isolated from brackish ecological niches (e.g. *R. violaceae* CCAP 1388/6; *D. polymorpha* CCAP 19/14; *C. cryptica* CCAP 1070/2 and *C. vulgaris* CCAP 211/21A), but thrived at seawater salinity levels (Supplementary Dataset S1 online). Overall, the common factors predisposing an individual taxon towards commercial exploitation seemed to be a high degree of adaptability and capacity for robust growth.

Micro-algae have received much interest as a source of high value omega-3 long-chain PUFA, for use in dietary supplements (i.e. valuable in commanding high commercial premium and health value), or for sequestering in the food chain in aquaculture or fisheries (i.e. of dietary health value added to the end product). Desaturated FA levels were altogether high in the screen compared with many terrestrial plant seed oils, and new strains for value-FA were noted^20^. For instance EPA productivity in *C. cryptica* CCAP 1070/2 matched that of a bench-mark *M. subterranea* CCAP 848/1 strain, used in aquaculture (Table 5)^35^. High DHA productivity was confined to the haptophytes, with 20 included in the screen. A routinely employed mariculture strain, *T. lutea* CCAP 927/14, emerged as the most productive, but a previously unstudied *Pavlova salina* CCAP 940/3 strain was also identified as a source of balanced EPA/DHA, with high TFA content. Overall, there was an inverse relationship noted between PUFA or total omega-3 long-chain PUFA levels versus TFA content, an observation which has previously been attributed to flux competition in FA biosynthesis^1^,^48^. Strains productive in omega-3 long-chain PUFA were often productive for protein, perhaps related to a reduced carbon partitioning into lipids (Table 5).

In order to maximize production rates of a desired product, a balance must be struck between partitioning of resources between its accumulation and cell growth^49^. Although noted in a smaller screen, there was no inverse correlation seen between TFA content and biomass productivity^50^. However, an inverse relationship was observed between biomass production and protein or N-content. In effect, the majority of the high biomass producers allocated most of their C into either carbohydrate or TFA by stationary phase, as opposed to protein or other organic N compounds, leading to high C/N ratios. Strains grown on relatively low-N media (saline f/2 or freshwater JM) might have undergone N-limitation and the most productive ones appeared to assimilate most of the supplied N into biomass, although there was some variation in this respect: *R. violaceae* CCAP 1388/6 and *C. cryptica* CCAP 1070/2 assimilated the most. Given that the cultures did not receive CO_2_ supplementation it is also plausible that some became C-limited which could in turn place energetic restrictions on N-assimilation^10^,^51^. Proteonomic/transcriptomic studies suggest that in oleaginous micro-algae, catabolic processes linked to down-regulation of photosynthesis at stationary phase are likely to contribute to non-polar lipid accumulation and recycling of organic N^52^,^53^,^54^. Taken together, a greater understanding of these processes is likely to benefit lipid production or N-remediation by algae, and requires further study in the high producer strains identified here.

To summarize, a comprehensive screen was undertaken and this provided a rapid, “intelligent” strategy for future high-throughput screening based on a primary elemental analysis step for identifying high TFA and biomass-producing algae. A detailed analysis of composition cast light on the partitioning of resources in algae and provided a data resource for comparative genomics methodology. A repertoire of model strains for further investigation of biofuels and bioremediation has been provided and these may be tested by up-scaling for biotechnological purposes.

## Methods

### Growth of Micro-algae

All micro-algae tested were from the CCAP, UK; www.ccap.ac.uk. Cultures were grown in a defined, artificial seawater-based medium (f/2), with the exception of 11 freshwater taxa from the genera: *Haematococcus*, *Dictyosphaerium* and *Cyanophora,* which were grow in JM and three freshwater taxa from the genera *Monodopsis*, *Eustigmatos* and *Desmodesmus* grown in 3NBBM+V (www.ccap.ac.uk). The f/2-based medium was prepared as follows: 33.5 gl^−1^ Instant Ocean (Aquarium Systems, France) pH adjusted to 6.9-7.0; the following added to final conc. 75 mgl^−1^ NaNO_3_, 5.65 mgl^−1^ NaH_2_PO_4_.2H_2_O, trace metals (final conc. Na_2_EDTA 4.16 mgl^−1^, FeCl_3_.6H_2_O 3.15 mgl^−1^, CuSO_4_.5H_2_O 0.01 mgl^−1^, ZnSO_4_.7H_2_O 0.022 mgl^−1^, CoCl_2_.7H_2_O 0.01 mgl^−1^, MnCl_2_.4H_2_O 0.18 mgl^−1^, Na_2_MoO_4_ .2H_2_O 0.006 mgl^−1^); in the case of diatoms 30 mgl^−1^ Na_2_SiO_3_.9H_2_O; Tris-base to 1 mM, pH adjusted to 6.8-7.0; vitamins (final conc. Cyanocobalamin 0.5 μgl^−1^, Biotin 0.5 μgl^−1^; Thiamine-HCl 0.1 mgl^−1^) were added after autoclaving. Growth was monitored by dual measurement of *in vivo* chlorophyll fluorescence and cell turbidity as described previously^43^. In the primary screen cultures of 100 mL were inoculated from starter cultures and incubated without agitation under a 12 h:12 h L/D (light/dark cycles) regime at 50-80 μmol m^−2^ sec^−1^ at 20 °C for 7-14 d, (Innova 44, New Brunswick Scientific, Edison, NJ). Cultures with no substantive growth after 14 d were discarded; cultures showing growth were used to inoculate secondary screen cultures once A_735_ = 0.34, or when chlorophyll fluorescence reached 10,000 RFU (Relative Fluorescence Units). These values equated to 1 × 10^7^ cells ml^−1^ of the standard model strain, *Nannochloropsis oculata* CCAP 849/1. Triplicate 400 mL cultures were inoculated at 5% (v/v) from starters into 500 mL aerated flasks as described^43^. Each flask was exposed to PAR (400-700 nm) 150 μmol photons m^−2^ s^−1^ for 16 h: 8 h L/D, at 20 °C throughout. A further 8 strains from the Rhodophyceae, the Porphyridiaceae and Dinophyceae were exposed to 50 μmol photons m^−2^ s^−1^; requiring lower light-levels^27^ and 4 polar diatom species required temperatures of 4-10 °C. Samples were harvested by centrifugation at 1000-4000 g, for 15 min (Sigma 4K15 centrifuge). *Dunaliella* species required 1000 g to avoid risk of cell rupture, whereas the Eustigmatophytes required 4000 g due to small-cell size. Harvested cells were then flash-frozen in liquid N, freeze-dried and stored as described^43^. Log phase samples (100 mL) were harvested based on the above biomass concentration proxies; DW biomass yields were later checked to be within 20-60% of stationary phase biomass. Once the cultures reached stationary phase the remainder of the culture was harvested (defined by no change >±5% in either A_735_ or chlorophyll fluorescence within a 2 d interval).

### Measurement of biomass and its constituents

Total C, N were determined by elemental analysis on 2 mg of freeze-dried material, and protein was determined by hot-TCA extraction followed by Lowry assay, as described^55^. TFA content was estimated by direct-derivatization of free and esterified FA by GC-FID as described (internal standards 10 μL 5 gl^−1^ tritricosanoin in chloroform or 100 μL 0.25 gl^−1^ 15:0 tripentadecanoin in hexane, Larodan, Malmö, Sweden)^43^. Individual FA were identified using a combination of internal standards and GC-MS analysis of FAMES and DMOX-derivatives in representative strains as described^43^. Total carbohydrate content was estimated using the Dubois assay^56^. Lyophilized 5 mg samples were suspended in 0.5 mL 1 M H_2_SO_4_ and extracted at 121 °C for 15 min. Samples were cooled and centrifuged for 10 min, 10,000 g. Assay was carried out on 10 μL supernatant by addition with gentle mixing of 0.5 mL of 4% phenol followed by 2.5 mL of conc. H_2_SO_4._ Readings were at A_490_, calibrating the assay with glucose.

### Data analysis

Compositional data for N, C, carbohydrate, protein, TFA and value FA were expressed in terms of biomass content (%DW), culture yield (gl^−1^), batch culture productivity from inoculation to harvest (gl^−1^d^−1^) and in the case of the specific FA, composition (%area). The best strains were ranked in excel for content, yield and productivity parameters, retaining strains above the 70^th^ percentile and comparisons of data by t-test. Graphical data output and Pearson’s correlations were carried out using PAST^57^ and statistical comparisons by 1-way ANOVA and MANOVA were carried out in MINITAB. The complete FA composition data-set was expressed as mol% using a cut-off of 0.1% prior to a hierarchical cluster analysis in PAST using rho-parameters. Phylogenetic analyses on 18S rDNA and ITS sequences were carried out using the Geneious 6.0.6 software package. Sequences were aligned using MUSCLE, editing out large insertions and drawing the trees using PhyML, bootstrapping for maximum likelihood inference where N = 1000.

## Additional Information

**How to cite this article**: Slocombe, S. P. *et al*. Unlocking nature's treasure-chest: screening for oleaginous algae. *Sci. Rep.* **5**, 09844; doi: 10.1038/srep09844 (2015).

## Supplementary Material

Supplementary Information

Supplementary Information

Supplementary Information

Supplementary Information

Supplementary Information

Supplementary Information

Supplementary Information

Supplementary Information

## Figures and Tables

**Table 1:** Top 26 micro-algal biomass producers. All strains were grown on f/2 unless denoted

**No.**	**Species**	**Strain**	**C productivity**		**C yield**		**C content**	
		**CCAP No.**	**(gl**^**−1**^**d**^**−1**^)	**SD**	**(gl**^**−1**^)	**SD**	**%DW**	**SD**
1	Monodopsis subterranea*	848/1	0.0163†	0.0039	0.244†	0.058	40.4	14.3
2	*Nannochloropsis gaditana*	849/5	0.0162†	0.0021	0.259†	0.034	51.3	3.8
3	*Nannochloropsis oceanica*	849/10	0.0155‡	0.0007	0.264†	0.012	63.0‡	0.5
4	*Nannochloropsis oceanica*	211/46	0.0148†	0.0003	0.237	0.004	58.7	1.3
5	*Dunaliella polymorpha*	19/14	0.0147†	0.0005	0.206	0.007	44.2	0.5
6	*Tetraselmis* sp.	66/60	0.0144†	0.0030	0.331†	0.068	44.0	1.9
7	*Cylindrotheca fusiformis*	1017/2	0.0138†	0.0014	0.194	0.020	47.1	1.0
8	*Nannochloropsis oceanica*	211/78	0.0133†	0.0016	0.212	0.026	58.2	1.5
9	*Dunaliella quartolecta*	19/8	0.0130	0.0006	0.209	0.010	43.1	1.1
10	*Nannochloropsis gaditana*	849/6	0.0128	0.0008	0.218	0.013	50.4	0.2
11	*Nannochloropsis oceanica*	849/8	0.0121	0.0017	0.206	0.029	52.5	2.5
12	*Chlorella vulgaris*	211/21A	0.0119	0.0004	0.263†	0.008	58.1	0.3
13	*Dunaliella quartolecta*	19/9	0.0119	0.0015	0.179	0.022	42.2	2.9
14	*Dunaliella tertiolecta*	19/22	0.0117	0.0005	0.223	0.009	44.3	0.6
15	*Tetraselmis apiculata*	66/15	0.0116	0.0005	0.254†	0.010	44.9	0.6
16	*Dunaliella bioculata*	19/4	0.0113	0.0002	0.181	0.003	43.9	0.7
17	*Nannochloropsis oceanica*	849/9	0.0113	0.0011	0.203	0.020	61.6†	3.1
18	*Dunaliella polymorpha*	19/7A	0.0111	0.0001	0.177	0.001	44.4	0.1
19	*Tetraselmis chui*	66/21A	0.0110	0.0015	0.243†	0.034	39.6	4.8
20	*Dunaliella tertiolecta*	19/6B	0.0106	0.0009	0.169	0.014	44.9	0.4
21	*Rhodella violaceae*	1388/6	0.0102	0.0005	0.275‡	0.014	18.4	1.2
22	*Pleuro. pseudoroscoffensis*	961/3	0.0094	0.0027	0.167	0.053	38.7	6.0
23	*Tetraselmis verrucosa*	66/6	0.0090	0.0013	0.226†	0.033	44.0	0.9
24	*Nannochloropsis salina*	849/4	0.0090	0.0012	0.171	0.024	48.2	8.0
25	*Tetraselmis* sp.	66/62	0.0088	0.0010	0.247†	0.028	41.3	1.9
26	*Isochrysis* sp.	927/12	0.0087	0.0007	0.165	0.014	32.4	3.3

^(*)^ in which case 3NBBM+V was used. All data points are above the 70^th^ percentiles for the screen.

^‡^Significantly different (P < 0.05) from rest of screen except where denoted

^(†).^ Full data in Supplementary Dataset S4 online.

**Table 2:** Top TFA-producing micro-algal strains.

**No.**	**Species**	**Strain**	**Productivity**		**Yield**		**Content**	
		**CCAP No.**	**(gl**^**−1**^**d**^**−1**^)	**SD**	**(gl**^**−1**^)	**SD**	**%DW**	**SD**
1	*Nannochloropsis oceanica*	849/10	0.0131*	0.0002	0.222†	0.003	53.2*	2.0
2	*Nannochloropsis gaditana*	849/5	0.0115†	0.0014	0.184†	0.023	36.4	2.4
3	*Chlorella vulgaris*	211/21A	0.0106	0.0010	0.234*	0.021	51.8†	4.2
4	*Nannochloropsis oceanica*	211/46	0.0101	0.0010	0.162	0.016	40.1	2.8
5	*Nannochloropsis oceanica*	849/8	0.0097	0.0019	0.165	0.033	41.9	4.1
6	*Nannochloropsis oceanica*	211/78	0.0096	0.0011	0.153	0.018	42.1	1.3
7	*Nannochloropsis gaditana*	849/6	0.0088	0.0008	0.150	0.014	34.6	1.1
8	*Nannochloropsis oceanica*	849/9	0.0080	0.0009	0.143	0.016	43.6	4.3
9	*Nannochloropsis oculata*	849/1	0.0075	0.0002	0.098	0.003	33.0	0.7
10	*Cylindrotheca fusiformis*	1017/2	0.0072	0.0014	0.101	0.020	24.5	3.1
11	*Nannochloropsis salina*	849/4	0.0069	0.0000	0.131	0.001	36.8	2.0
12	*Tetraselmis sp.*	66/60	0.0065	0.0025	0.150†	0.058	19.5	4.7
13	*Extubocellulus spinifer*	1026/2	0.0059	0.0012	0.053	0.011	23.1	2.3
14	*Dunaliella primolecta*	11/34	0.0054	0.0003	0.081	0.005	31.9	2.8
15	*Nannochloropsis salina*	849/2	0.0052	0.0008	0.099	0.015	36.8	4.6
16	*Chaetoceros muelleri*	1010/3	0.0052	0.0021	0.078	0.032	23.9	2.9
17	*Chaetoceros simplex*	1085/3	0.0052	0.0015	0.067	0.019	19.6	3.0
18	*Nannochloropsis oculata*	849/7	0.0050	0.0004	0.085	0.007	36.7	3.4
19	*Chlorella stigmatophora*	211/20	0.0049	0.0001	0.102	0.003	30.7	3.0
20	*Haematococcus pluvialis*‡	34/6	0.0047	0.0016	0.167†	0.052	36.4†	10.6
21	*Chlorella vulgaris*	211/75	0.0047	0.0026	0.102	0.057	29.9	9.2
22	*Chlorella sp.*	211/53	0.0043	0.0006	0.107	0.014	24.5	1.2
23	*Cyclotella cryptica*	1070/2	0.0040	0.0005	0.059	0.007	23.5	3.2
24	*Nannochloropsis salina*	849/3	0.0038	0.0023	0.069	0.042	36.8	6.5
25	*Chloroidium saccharophilum*	211/27	0.0028	0.0006	0.079	0.016	20.9	2.0
26	*Haematococcus pluvialis*‡	34/1F	0.0027	0.0005	0.094	0.017	26.5	3.2

^*^Significantly different (P < 0.05) from rest of column except where denoted

^(†).^All data points are above the 70^th^ percentiles for the screen. All strains were grown on f/2 unless denoted

^(‡)^where JM was used. Full data set in Supplementary Dataset S4 online.

**Table 3:** Top producing strains for protein, N and carbohydrate.

**No.**	**Species**	**Strain**	**Productivity**		**Yield**		**Content**	
		**CCAP No.**	**(gl**^**−1**^**d**^**−1**^)	**SD**	**(gl**^**−1**^)	**SD**	**%DW**	**SD**
Protein								
1	Monodopsis subterranea*	848/1	0.0057†	0.0015	0.0856†	0.0227	13.6	1.83
2	Eustigmatos vischeri*	860/7	0.0040‡	0.0008	0.0598‡	0.0122	20.2‡	5.07
3	*Tisochrysis lutea*	927/14	0.0036‡	0.0004	0.0501‡	0.0053	15.3‡	1.07
4	*Chroomonas placoidea*	978/8	0.0033‡	0.0002	0.0465	0.0024	13.8	0.40
5	*Chaetoceros muelleri*	1010/3	0.0028	0.0008	0.0417	0.0113	13.1	2.56
6	*Pleurochrysis dentata*	904/1	0.0026	0.0003	0.0440	0.0056	14.1	1.81
7	*Chlorella vulgaris*	211/75	0.0025‡	0.0008	0.0557‡	0.0180	14.6‡	1.68
8	*Rhodomonas reticulata*	995/2	0.0024	0.0001	0.0342	0.0019	19.3†	2.46
9	*Cyclotella cryptica*	1070/2	0.0023	0.0001	0.0349	0.0020	13.8	1.01
10	*Pycnococcus provasolii*	190/1	0.0022	0.0003	0.0359	0.0050	15.7‡	2.04
N								
1	Monodopsis subterranea*	848/1	0.0025†	0.0005	0.0371†	0.0069	6.1‡	1.8
2	Eustigmatos vischeri*	860/7	0.0012	0.0002	0.0186	0.0036	6.4‡	2.0
3	*Extubocellulus spinifer*	1026/2	0.0010	0.0000	0.0093	0.0004	4.1	0.3
4	*Cyclotella cryptica*	1070/2	0.0010	0.0000	0.0148	0.0006	5.9	0.3
5	Desmodesmus elegans*	258/8	0.0009	0.0002	0.0335‡	0.0082	8.9†	0.1
6	*Pycnococcus provasolii*	190/1	0.0007	0.0000	0.0116	0.0004	5.1	0.5
7	*Pavlova salina*	940/3	0.0007	0.0000	0.0092	0.0002	4.2	0.5
*Carbohydrate*								
1	*Dunaliella polymorpha*	19/14	0.0203†	0.0007	0.2841‡	0.0103	61.0	2.42
2	*Dunaliella quartolecta*	19/8	0.0170	0.0012	0.2723‡	0.0191	56.4	4.65
3	*Dunaliella tertiolecta*	19/22	0.0167	0.0012	0.3181†	0.0224	63.2	4.23
4	*Dunaliella quartolecta*	19/9	0.0151	0.0009	0.2261	0.0134	53.6	5.49
5	*Dunaliella bioculata*	19/4	0.0143	0.0002	0.2286	0.0037	55.5	1.07
6	*Tetraselmis chui*	66/21A	0.0141	0.0017	0.3113‡	0.0383	50.8	4.82
7	*Dunaliella polymorpha*	19/7A	0.0139	0.0008	0.2224	0.0135	55.8	3.71
8	*Dunaliella tertiolecta*	19/6B	0.0137	0.0017	0.2197	0.0270	58.2	1.72
9	*Dunaliella salina*	19/12	0.0125	0.0019	0.1880	0.0283	47.4	3.92
10	*Haematococcus pluvialis*§	34/6	0.0118‡	0.0065	0.4166‡	0.2183	81.1*	7.89

All strains were grown on f/2 unless denoted (*) where 3NBBM+V was used or (§) where JM was used.

^†^Significantly different (P < 0.05) from rest of the column except where denoted (‡).

All data points are above the 70^th^ percentiles for the screen.

Full data set in Supplementary Dataset S4 online.

**Table 4:** Strains producing high-value omega-3 long-chain PUFA.

**No.**	**Species**	**Strain**	**Specified FA Productivity**		**Specified FA Yield**		**Specified FA composition**		**Specified FA content**		**TFA content**	
		**CCAP No.**	**(mgl-d^-1^)**	**SD**	**(mgl^-1^)**	**SD**	**%Area**	**SD**	**%DW**	**SD**	**%DW**	**SD**
EPA												
1	Monodopsis subterranea*	848/1	0.77†	0.09	11.48†	1.40	22.5†	0.6	1.9	0.4	8.4	1.9
2	*Cyclotella cryptica*	1070/2	0.63‡	0.04	9.50‡	0.60	16.1	0.8	3.8†	0.3	23.5†	3.2
3	Eustigmatos vischeri*	860/7	0.53	0.11	7.91	1.59	22.2‡	1.9	2.6	0.4	11.8	1.2
4	*Pavlova salina*	940/3	0.44	0.08	6.21	1.11	14.3	0.3	2.9‡	0.6	20.0‡	4.2
5	*Nitzschia ovalis*	1052/12	0.41	0.13	3.30	1.07	15.4	0.8	3.1‡	0.4	20.4‡	3.2
6	*Thalassiosira weissflogii*	1085/18	0.36	0.07	5.77	1.05	13.4	0.2	1.7	0.1	12.3	1.2
7	*Pavlova pinguis*	940/2	0.33‡	0.16	7.60	3.59	15.6	0.9	3.3‡	1.6	21.4‡	9.9
8	*Pavlova gyrans*	940/1B	0.31	0.01	6.01	0.47	14.7	0.8	1.8	0.1	12.5	1.1
9	*Diacronema lutheri*	931/7	0.29	0.15	3.79	1.94	16.4	1.1	2.2	0.5	13.7	3.9
10	*Thalassiosira weissflogii*	1085/1	0.27	0.02	5.77	0.51	11.4	0.9	1.8	0.1	15.5	1.0
11	*Thalassiosira pseudonana*	1085/12	0.25	0.14	3.86	1.51	10.4	1.5	2.2	0.8	21.4‡	6.3
12	*Nitzschia epithemoides*	1052/18	0.19	0.03	4.27	0.72	13.9	1.8	2.6	0.3	19.2‡	3.4
DHA												
1	*Tisochrysis lutea*	927/14	0.44†	0.03	6.10‡	0.4	16.4†	0.3	1.9	0.1	11.4	0.6
2	*Prymnesium parvum*	946/4	0.41‡	0.09	8.91†	2.0	14.3	0.5	2.3‡	0.3	16.1‡	2.1
3	*Isochrysis* sp.	927/12	0.38‡	0.03	7.15‡	0.6	11.9	0.1	1.4	0.4	12.1	3.4
4	*Chrysotila lamellosa*	918/1	0.35	0.04	5.96‡	0.7	9.1	1.6	1.2	0.1	13.7‡	1.3
5	*Prymnesium parvum*	946/6	0.29‡	0.13	5.47‡	2.7	13.0	1.6	3.4†	0.9	26.7†	8.5
6	*Isochrysis galbana*	949/1	0.23	0.08	3.97	1.4	10.7	0.8	1.3	0.2	11.9	1.1
7	*Pedinella marina*	941/1A	0.23‡	0.13	5.05‡	3.0	18.3‡	7.5	1.9‡	1.2	9.8	2.2
8	*Isochrysis galbana*	927/1	0.23	0.03	4.52	0.6	13.4	0.2	2.1	0.1	16.0‡	1.1
9	*Diacronema lutheri*	931/7	0.22‡	0.14	2.89	1.8	11.9	2.1	1.7‡	0.6	13.7‡	3.9
10	*Pedinella* sp.	941/3	0.21	0.02	2.99	0.3	11.4	0.5	1.5	0.2	13.5‡	1.7
11	*Pavlova salina*	940/3	0.18	0.03	2.57	0.4	5.9	0.1	1.2	0.3	20.0‡	4.2
12	*Pleuro. pseudoroscoffensis*	913/2	0.18	0.03	2.89	0.5	8.3	0.2	0.9	0.1	11.2	1.2
13	*Pleurochrysis carterae*	961/4	0.17	0.01	2.80	0.2	8.6	0.6	1.1	0.1	12.9	1.3
14	*Pleurochrysis dentata*	904/1	0.15	0.02	2.57	0.4	5.9	0.8	0.8	0.1	14.4‡	3.4
15	*Pleurochrysis pinguis*	940/2	0.13	0.06	2.92	1.5	6.0	0.6	1.3	0.7	21.4‡	9.9

All strains were grown on f/2 unless denoted (*) where 3NBBM+V was used.

^†^Significantly different (P < 0.05) from rest of the column except where denoted (‡). All data are above the 70^th^ percentile for the screen for the first 4 parameters. Full data set in Supplementary Dataset S7 online.

**Table 5:** Summary of the most productive strains emerging from the screen.

**No.**	**Species**	**Biomass**	**TFA**	**Carbohydrate**	**Protein**	**EPA**	**DHA**	**SDA**	**GLA**	**Total ω-3 FA**	**N-assim.**	**Potential application/comment**
1	*Monodopsis subterranea* CCAP 848/1*	+++			+++	+++						Protein/EPA-rich feeds/aquaculture
2	*Nannochloropsis gaditana* CCAP 849/5	+++	+++									Biofuels: TFA 36%DW
3	*Nannochloropsis oceanica* CCAP 849/10†	+++	+++									Biofuels: TFA >50%DW
4	*Nannochloropsis oceanica* CCAP 211/46	+++	+++									Biofuels: TFA 40%DW
5	*Dunaliella polymorpha* CCAP 19/14	+++		+++								Bioethanol/gas: carbohydrate >60%DW
6	*Tetraselmis* sp. CCAP 66/60‡	+++	++								+	Mariculture/feeds: balanced composition
7	*Cylindrotheca fusiformis* CCAP 1017/2	++	++						+++			Biofuel/Model for C_14_ biofuel, flocculates
8	*Nannochloropsis oceanica* CCAP 211/78†	++	+++									Biofuels: TFA 42%
9	*Chlorella vulgaris* CCAP 211/21A	+	+++							+++		Biofuel/feeds: TFA>50% DW, ALA 13% TFA
10	*Rhodella violaceae* CCAP 1388/6	+									+	Biogas/N-remediation. High DW yields
11	*Cyclotella cryptica* CCAP 1070/2		+		+	+++					+	EPA source (16% in TFA): TFA 24% DW
12	*Eustigmatos vischeri* CCAP 860/7*				+++	+++						Protein/EPA-rich feeds: protein 20% DW
13	*Isochrysis* sp. CCAP 927/12						+++	++				Mariculture/feeds: DHA-rich
14	*Tisochrysis lutea* CCAP 927/14				+++		+++	+++		+++		Mariculture/feeds: ω-3’s, protein 15%DW
15	*Prymnesium parvum* CCAP 946/4			+			+++	++		++		DHA source (14% in TFA): TFA 16% DW
16	*Pavlova salina* CCAP 940/3					++	++		+			EPA/DHA (14%/6% in TFA): TFA 20% DW
17	*Chroomonas placoidea* CCAP 978/8				+++			+++		+++		Mariculture/feed: protein 14% DW
18	*Dictyo. ehrenbergianum* CCAP 222/1A*								+++			GLA source (6% in TFA): TFA 17%DW
19	*Dictyo. ehrenbergianum* CCAP 222/1C*									+++		Aquaculture: ω-3 long-chain PUFA rich
20	*Pleurochrysis dentata* CCAP 944/2							+++				SDA source (11% in TFA): TFA 18% DW

Strains are arranged in descending order of biomass productivity (gC l^−1^ d^−1^) focussing on best strains for a given species/genus. Scoring system refers to productivity: >95^th^ percentile (+++); >90^th^ percentile (++) and >70^th^ percentile (+) except for N-assimilation where this indicates high assimilation of supplied N. All tested under full-salinity culture unless indicated (*) where freshwater.

^†^Commercial origin (see Supplementary Dataset S1 online). Full data found in Supplementary Dataset S4-S7 online. ω-3’s: omega-3 long-chain PUFAs.
