# Supplementary material for: Unlocking nature’s treasure-chest: screening for oleaginous algae
Source: Sci Rep. 2015 Jul 23;5:9844. doi: 10.1038/srep09844 (PMC5378892; doi:10.1038/srep09844)
Supplement: Supplementary Information [file srep09844-s1.pdf]

Supplementary Information

Unlocking nature's treasure-chest: screening for oleaginous algae

Stephen P. Slocombe[1]*, QianYi Zhang[1], Michael Ross[1], Avril Anderson[1], Naomi J. Thomas[1], Ángela Lapresa[1], Cecilia Rad Menéndez[2], Christine N. Campbell[2], Kenneth D. Black[3], Michele S. Stanley[1], John G. Day[2]

[1]Microbial and Molecular Biology Department, Scottish Association for Marine Science (SAMS), Scottish Marine Institute, Oban, PA37 1QA, UK

[2]Culture Collection of Algae and Protozoa (CCAP), Scottish Association for Marine Science (SAMS), Scottish Marine Institute, Oban, PA37 1QA, UK

[3]Ecology Department, Scottish Association for Marine Science (SAMS), Scottish Marine Institute, Oban, PA37 1QA, UK

*Correspondence should be directed to S.P.S (stephen.slocombe@sams.ac.uk)

Supplementary Information Table of Contents

SUPPLEMENTARY FIGURES S1-10 (pp 3-13)

Figure S1. Comparison of biomass evaluated by mass C and DW 3

Figure S2. Relationship between biomass TFA content and growth. 4

Figure S3. Molecular phylogeny of *Nannochloropsis* species 5

Figure S4. Relationship between protein content and N-content 6

Figure S5. Inverse relationship between N-content and productivity 7

Figure S6. Inverse relationship between protein content and productivity 8

Figure S7. Distribution of C/N ratios 9

Figure S8. Partitioning of resources in the top biomass producing strains 10

Figure S9. Fatty acid composition of micro-algae 11

Figure S10. Relationship between biomass TFA content and fatty acid desaturation 12

Figure S11. TFA productivity and the component due to omega-3 long-chain PUFA 13

SUPPLEMENTARY TABLES (pp 14-15)

Table S1.Top strains producing the value fatty acids SDA and GLA...................................... 14

Table S2. Strains producing high levels of total omega-3 long-chain PUFA 15

SUPPLEMENTARY TEXT (pp 16-23)

Text S1. FA composition 16

SUPPLEMENTARY REFERENCES (pp 24-25)

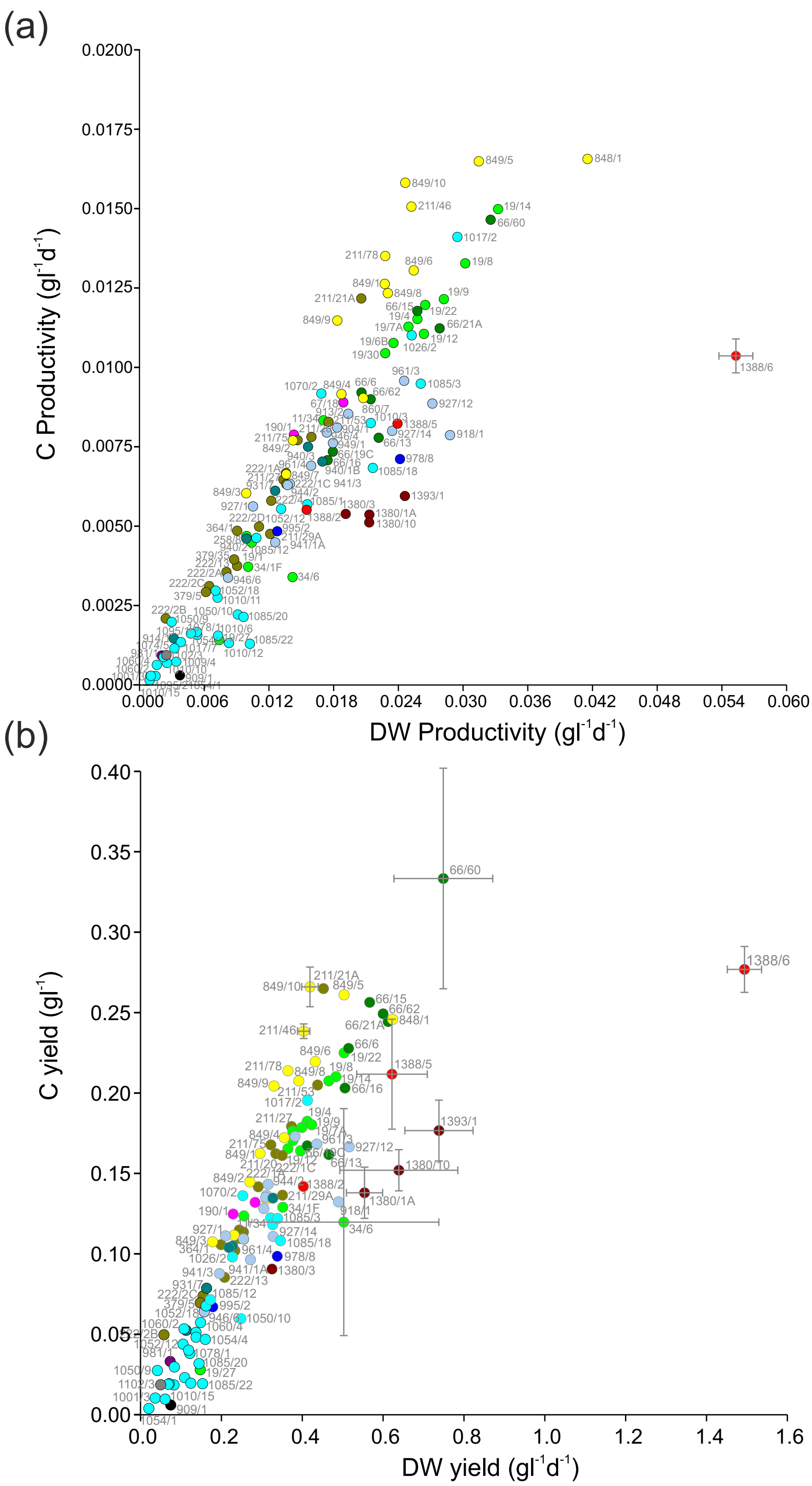

Figure S1. Comparison of biomass evaluated by mass C and DW. Data points for the entire secondary screen are shown for (a) biomass productivity and (b) biomass yield. Mass C was determined by elemental analysis. Error bars depicting SD are shown for the outliers. Data points are coloured according to class as depicted in Fig. 1. Labels refer to CCAP accession number.

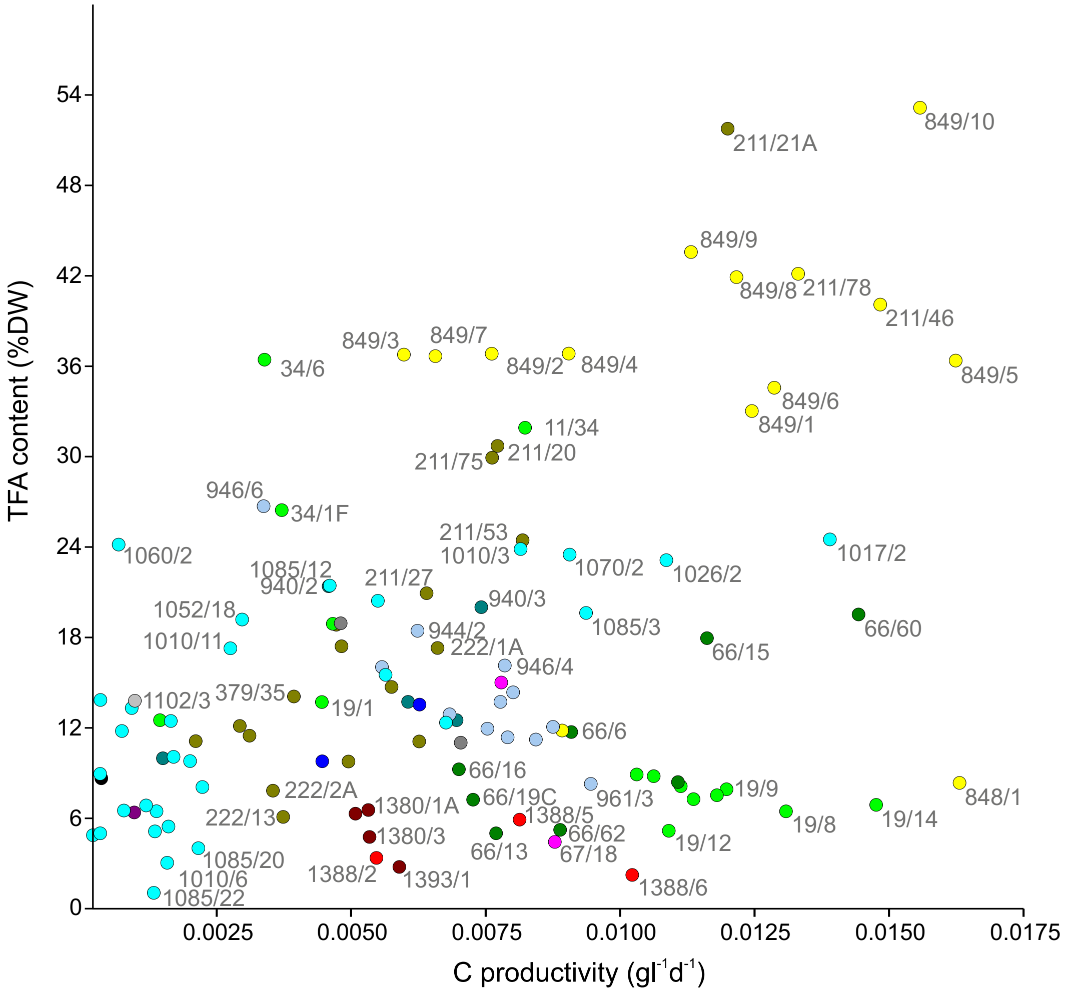

Figure S2. Relationship between biomass TFA content and growth. TFA content (%DW) was measured in terms of total FAME measured by GC-FID, and growth in terms of biomass C productivity, where mass C was determined by elemental analysis. Data points for the entire secondary screen are shown and are coloured according to class as depicted in Fig. 1. Labels refer to CCAP accession number.

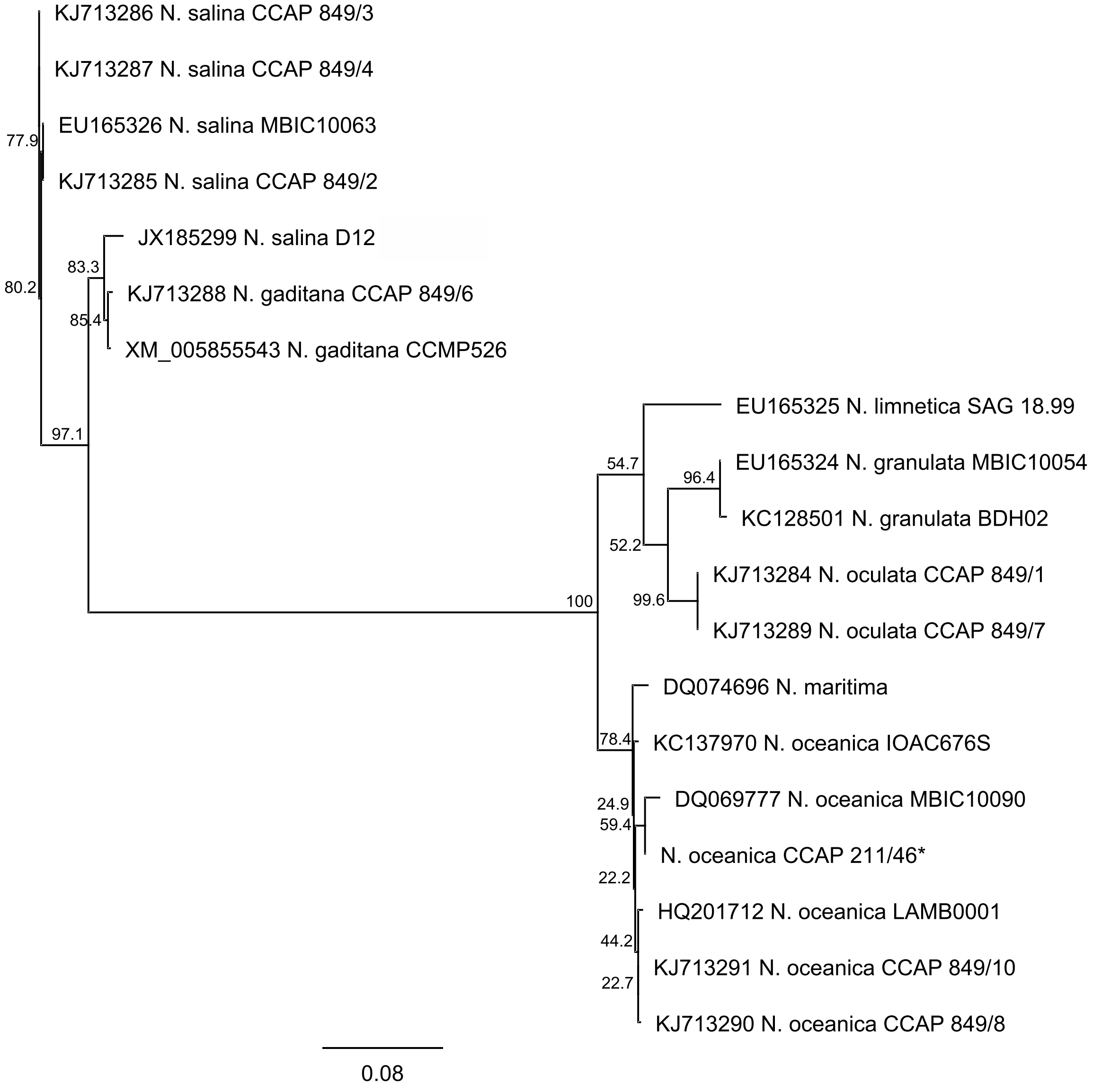

Figure S3. Molecular phylogeny of *Nannochloropsis* species. This was inferred from a comparison of ITS rDNA sequences. The tree depicted resulted from a maximum likelihood analysis based on an alignment encompassing ITS1, 5.8S rDNA and ITS2. Bootstrap percentage values are shown where N=1000.*Locus: nanno_4839:2492-3399 of the synonymous strain CCMP 1779 (https://benning-linux.bch.msu.edu/cgi-bin/gb2/gbrowse/Nannochloropsis_oceanica_CCMP1779_v1/).

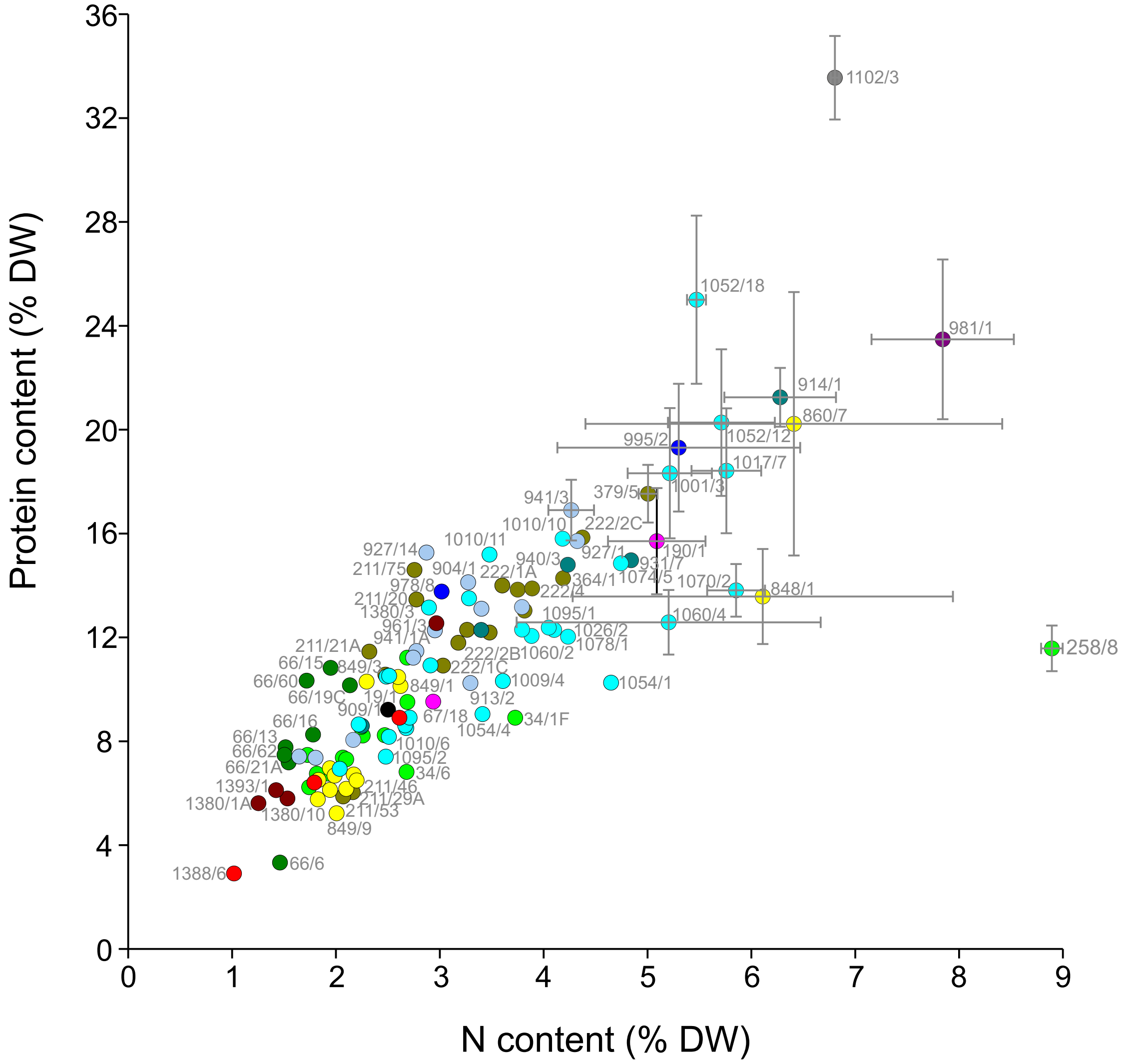

Figure S4. Relationship between protein content and N-content. Mass N (%DW) was determined by elemental analysis and protein content by Lowry assay. Error bars depicting SD are shown for the outliers and high content strains. Data points for the entire secondary screen are shown and are coloured according to class as depicted in Fig. 1.

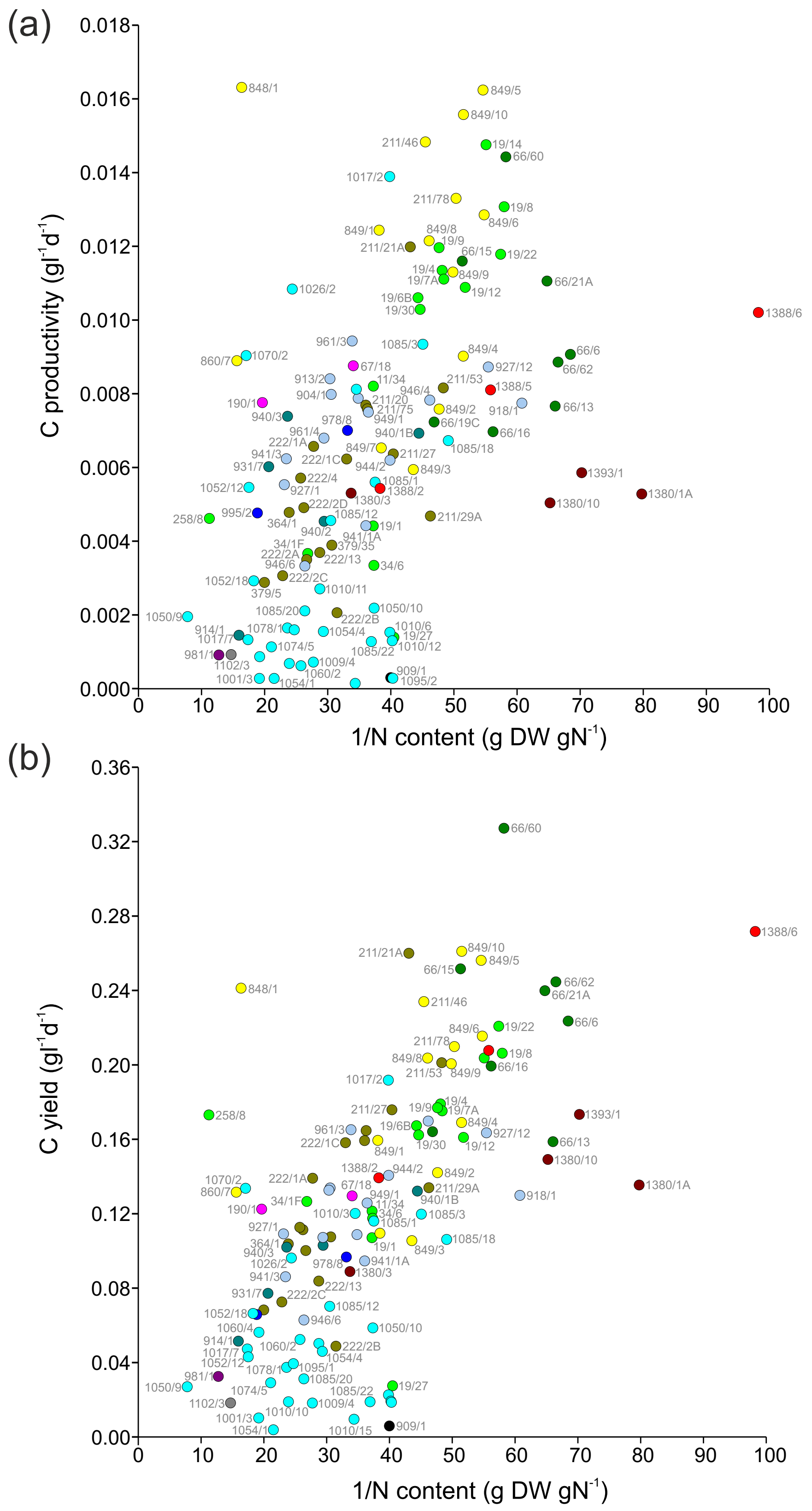

Figure S5. Inverse relationship between N-content and productivity. Mass N and C were determined by elemental analysis. The reciprocal of N content (%DW) is compared with (a) C productivity and (b) C yield. Data points for the entire secondary screen are shown and are coloured according to class as depicted in Fig. 1. Labels refer to CCAP accession number.

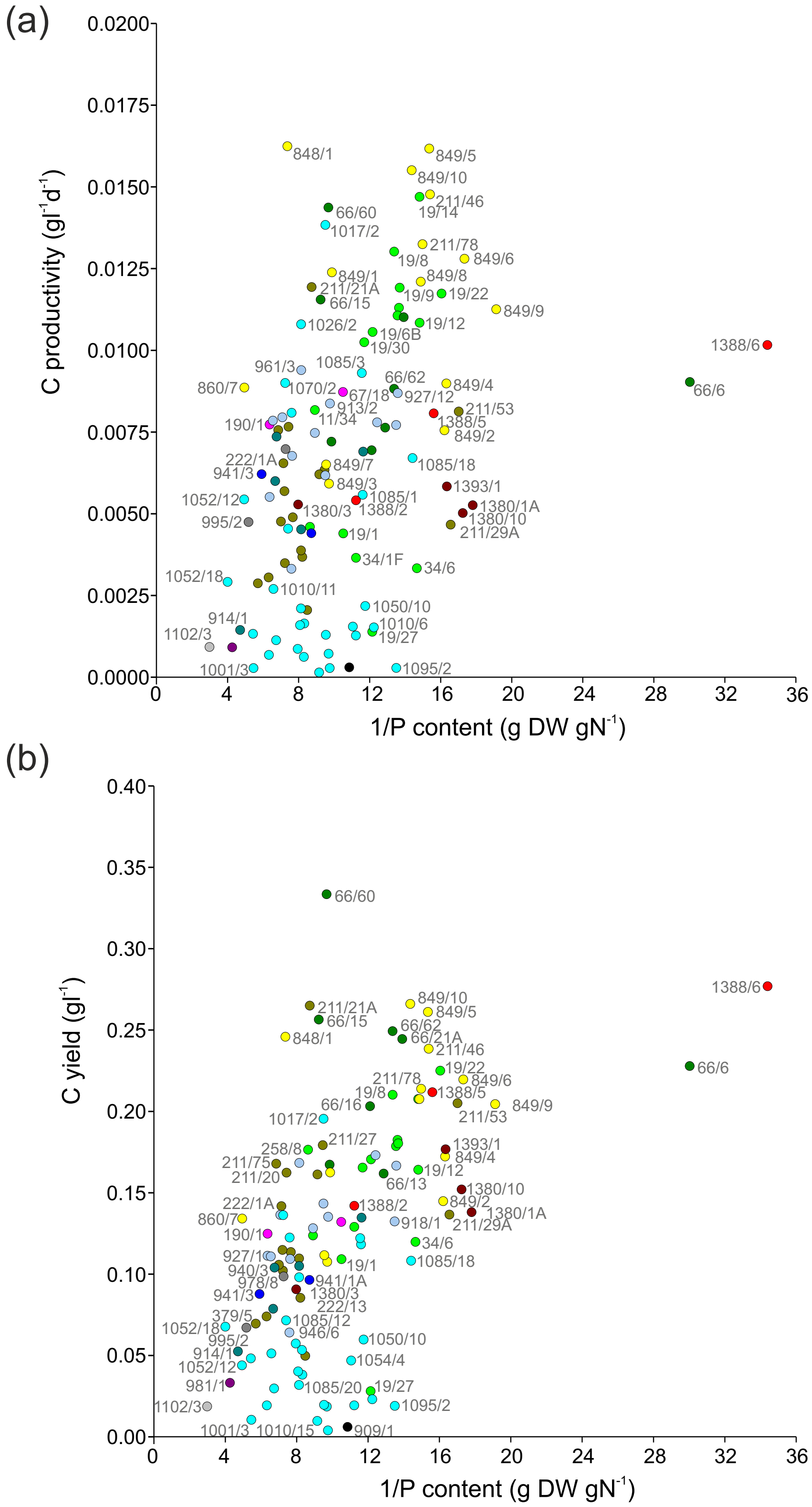

Figure S6. Inverse relationship between protein content and productivity. The reciprocal of protein content (%DW) measured by Lowry assay is compared with (a) C productivity and (b) C yield, measured by elemental analysis. Data points for the entire secondary screen are shown and are coloured according to class as depicted in Fig. 1. Labels refer to CCAP accession number.

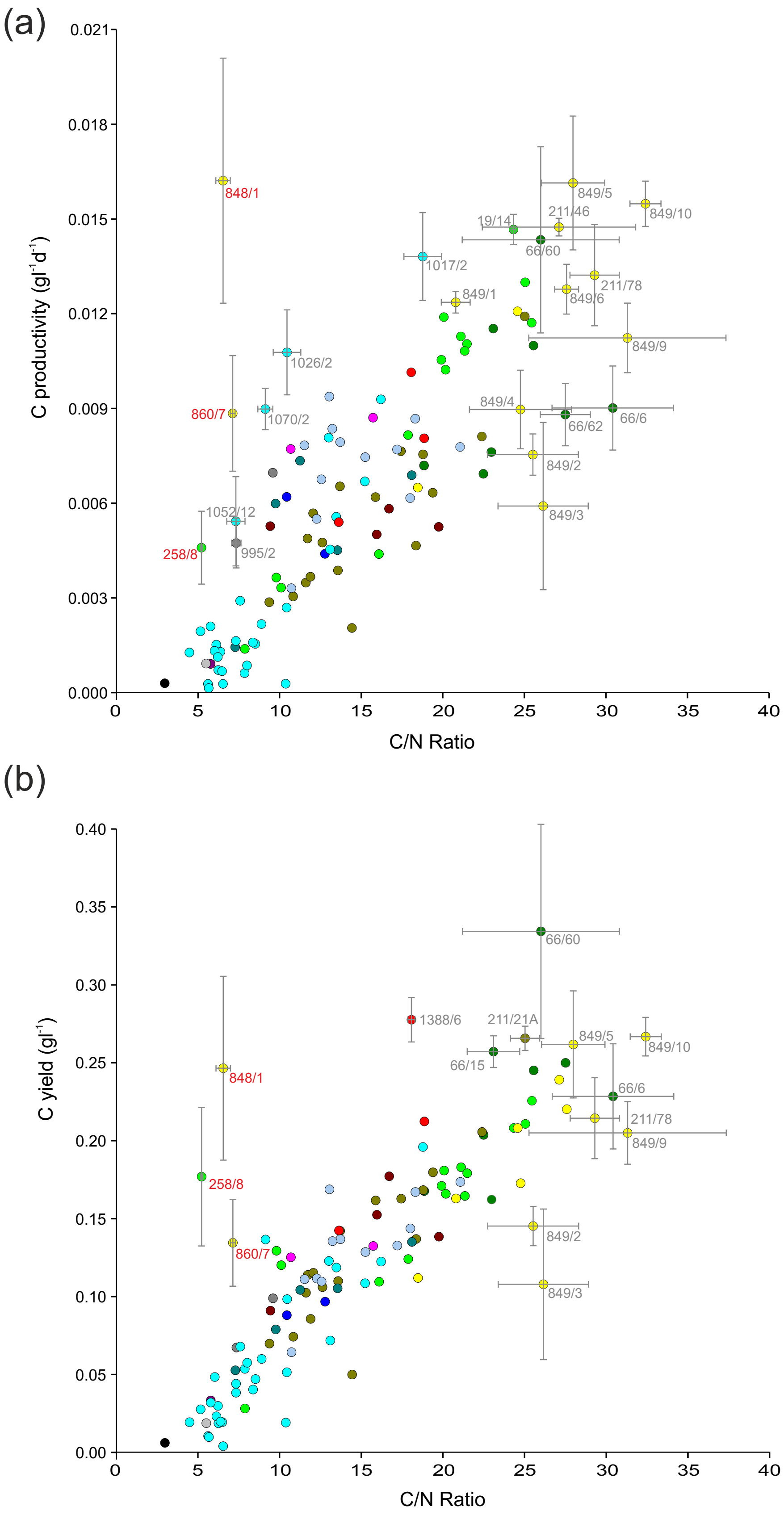

Figure S7. Distribution of C/N ratios. Mass N and C determined by elemental analysis is expressed in terms of C/N ratio and shown in relation to (a) C productivity and (b) C yield. Data points for the entire secondary screen are shown and are coloured according to class as depicted in Fig. 1. Error bars depict SD. Labels refer to CCAP accession number with strains grown in high-N media (3NBBM+V) indicated in red.

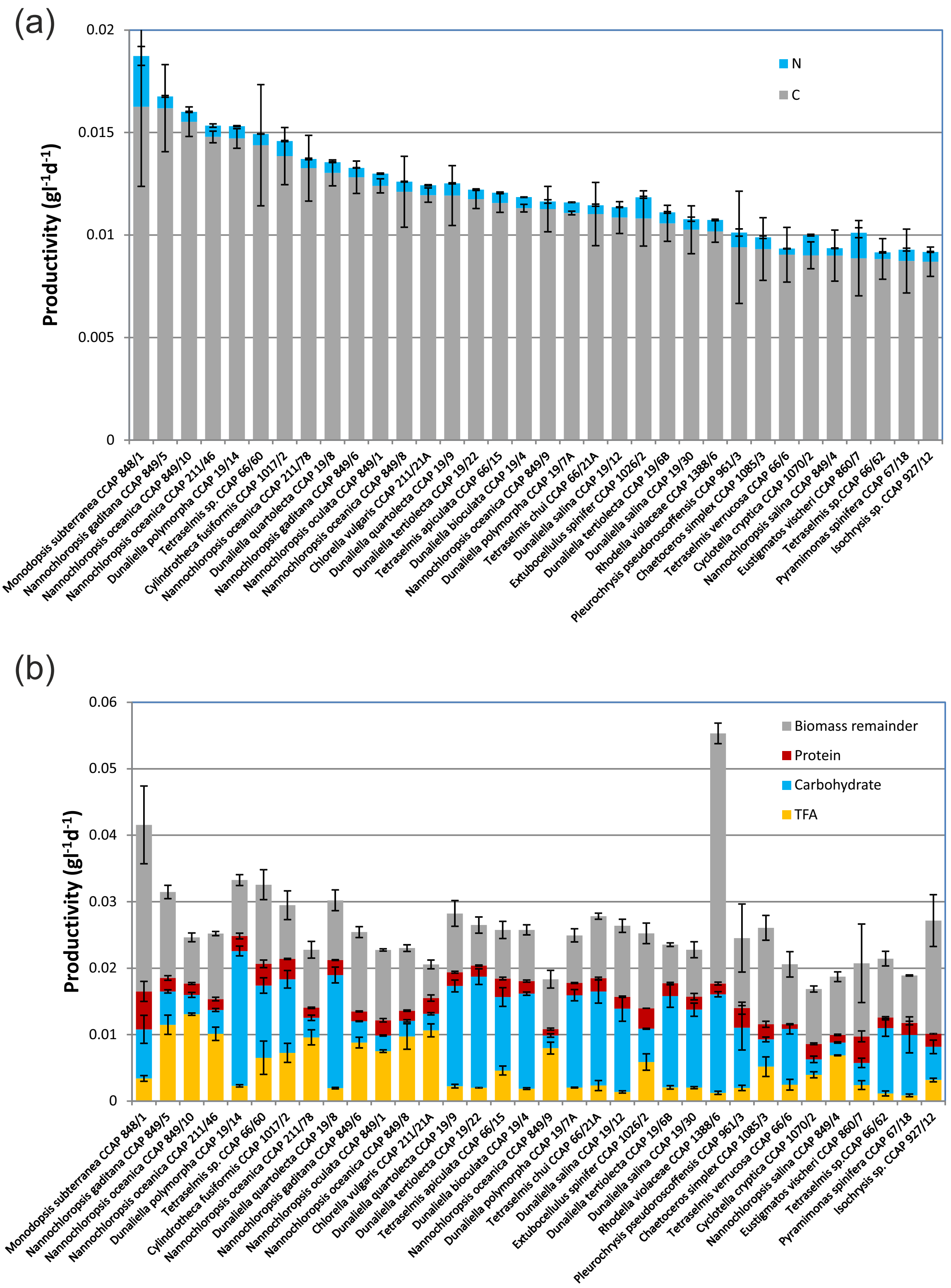

Figure S8. Partitioning of resources in the top biomass producing strains. (a) combined C and N productivities and (b) DW productivity subdivided into TFA, protein, carbohydrate and remaining biomass components. Data are ranked according to C productivities and these data are the 70th percentile for C productivity. Full data in Supplementary Dataset S4 online.

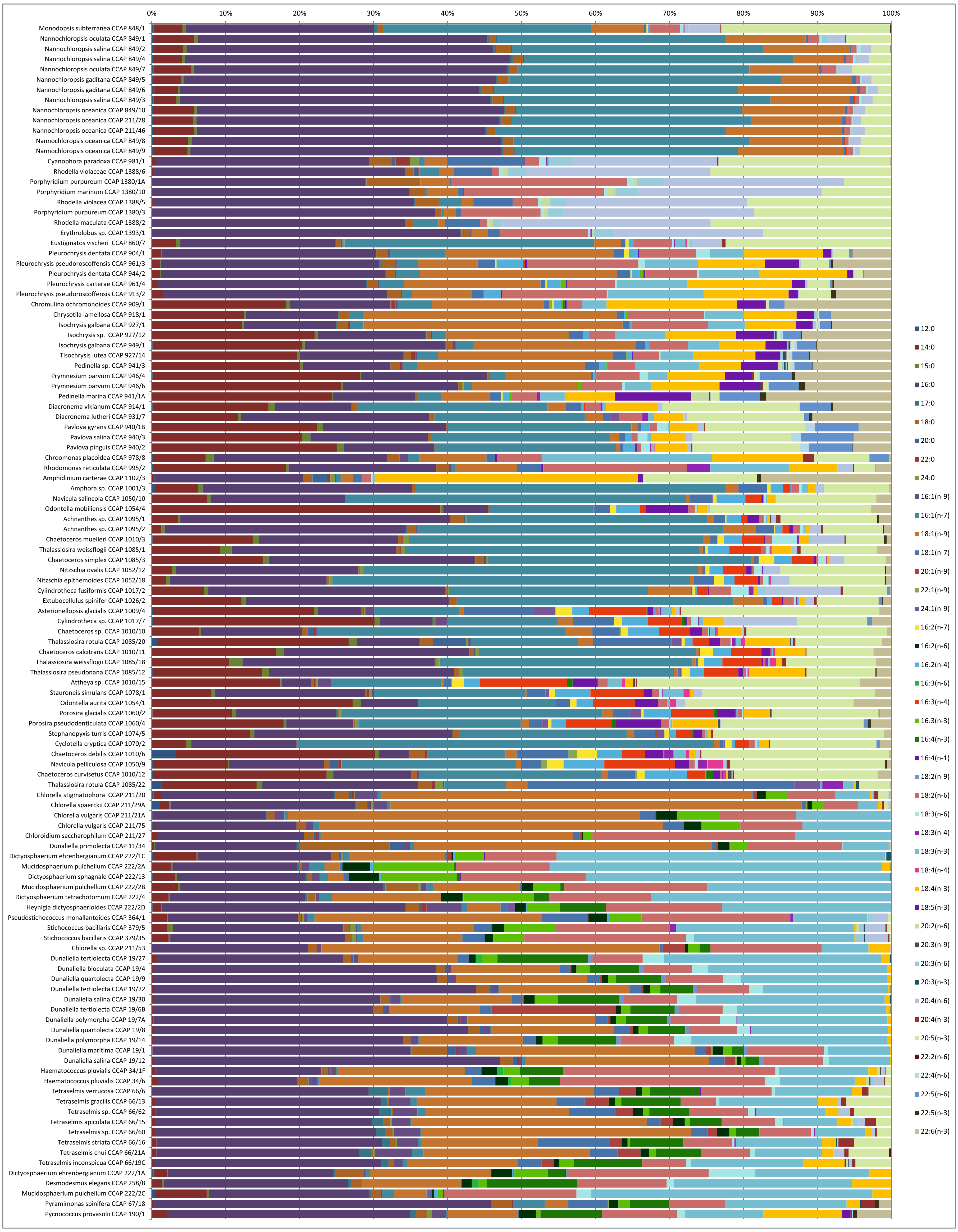

Figure S9. Fatty acid composition of micro-algae examined in the screen. Data are arranged graphically according to the hierarchical cluster depicted in Fig. 5. Compositional data are expressed as mol% and were subject to a 0.1% cut-off and clustered using a PAST algorithm with Rho-parameters. FA identities are indicated according to the colour scheme on the right. The data are also tabulated in Supplementary Dataset S6 online.

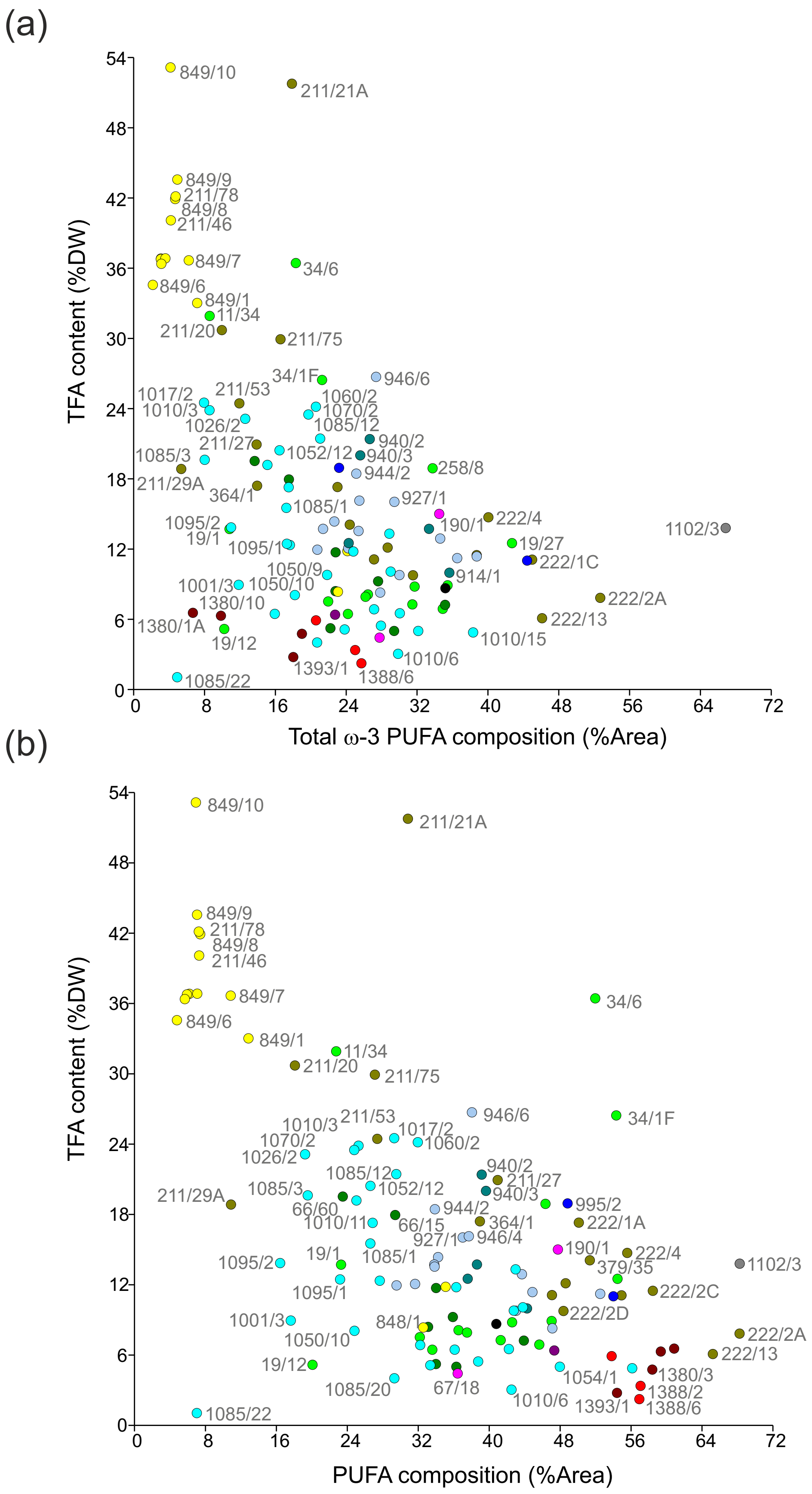

Figure S10. Relationship between biomass TFA content and fatty acid desaturation. Desaturation was expressed in terms of (a) total omega-3 long-chain PUFA content in TFA and (b) PUFA content in TFA on a %peak area basis. Data points for the entire secondary screen are shown and are coloured according to class as depicted in Fig. 1. Labels refer to CCAP accession number.

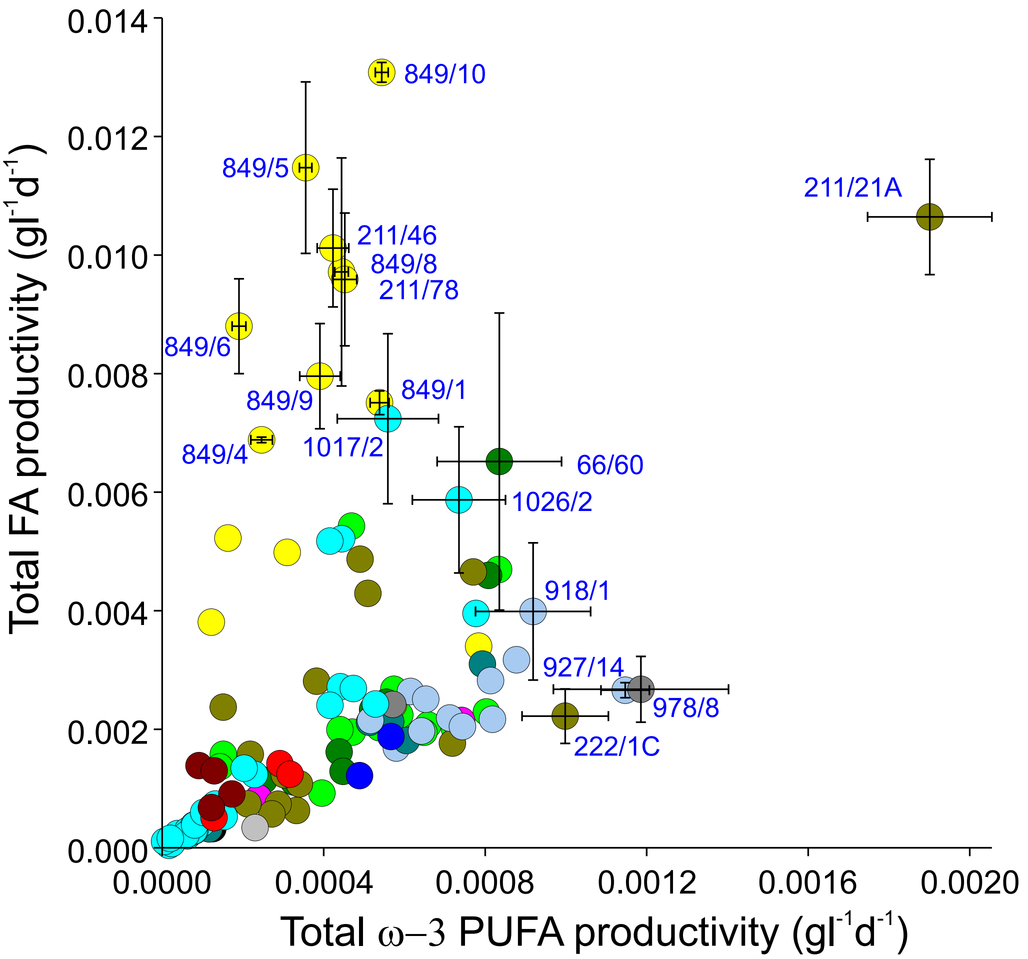

Figure S11. TFA productivity and the component due to omega-3 long-chain PUFA. Data points for the entire secondary screen are shown and are coloured according to class as depicted in Fig. 1. Error bars are shown for key algal strains which are labelled according to CCAP accession no. Data are tabulated in Supplementary Dataset S4 online.

SUPPLEMENTARY TABLES

Table S1 Top strains producing the high value fatty acids SDA and GLA

No.	Species	Strain CCAP No.	Specified FA Productivity (mgl^{-1}d^{-1})	SD	Specified FA Yield (mgl^{-1})	SD	Specified FA composition %Area	SD	Specified FA content %DW	SD	TFA content %DW	SD
SDA												
1	*Tisochrysis lutea*	927/14	0.45*	0.03	6.3*	0.4	16.9*	0.2	1.9*	0.1	11.4	0.6
2	*Chroomonas placoidea*	978/8	0.32	0.06	4.5	0.9	11.9	0.1	1.3	0.2	11.0	1.5
3	*Pleurochrysis dentata*	944/2	0.29	0.08	6.6†	1.8	11.4	1.6	2.1†	0.7	18.4†	4.5
4	*Pleurochrysis carterae*	961/4	0.27	0.02	4.3	0.3	13.1	0.3	1.7†	0.2	12.9†	1.3
5	*Chrysotila lamellosa*	918/1	0.26	0.05	4.4	0.8	6.7	1.0	0.9	0.1	13.7†	1.3
6	*Pleurochrysis dentata*	904/1	0.26	0.04	4.4	0.6	10.0	1.1	1.4	0.2	14.4†	3.4
7	*Isochrysis sp.*	927/12	0.25	0.04	4.7	0.7	7.7	0.6	0.9	0.3	12.1†	3.4
8	*Pleurochrysis pseudoroscoffensis*	913/2	0.23	0.04	3.7	0.6	10.7	0.3	1.2	0.2	11.2	1.2
9	*Pycnococcus provasolii*	190/1	0.22	0.03	3.5	0.4	10.3	0.7	1.5	0.2	15.0†	1.9
10	*Prymnesium parvum*	946/4	0.21	0.04	4.6	0.9	7.4	0.3	1.2	0.2	16.1†	2.1
11	*Thalassiosira pseudonana*	1085/12	0.20	0.14	3.01	1.7	7.7	2.3	1.7†	0.9	21.4†	6.3
12	*Prymnesium parvum*	946/6	0.19	0.07	3.62	1.5	8.8	0.3	2.3†	0.7	26.7*	8.5
13	*Pleuro. pseudoroscoffensis*	961/3	0.16	0.05	2.91	0.8	8.3	1.3	0.7	0.2	8.3	1.7
14	*Rhodomonas reticulata*	995/2	0.16	0.05	2.25	0.8	6.5	0.9	1.3	0.4	18.9†	3.0
15	*Isochrysis galbana*	927/1	0.15	0.02	3.03	0.4	9.0	0.4	1.4	0.2	16.0†	1.1
GLA												
1	*Chaetoceros muelleri*	1010/3	0.18*	0.08	2.70†	1.2	3.4	0.2	0.8†	0.1	23.9†	2.9
2	*Cylindrotheca fusiformis*	1017/2	0.18†	0.04	2.46†	0.6	2.4	0.1	0.6†	0.1	24.5†	3.1
3	*Dictyosphaerium ehrenbergianum*‡	222/1A	0.14†	0.04	3.07†	1.2	5.9*	0.9	1.0*	0.3	17.3†	2.3
4	*Haematococcus pluvialis*‡	34/6	0.09†	0.04	3.26*	1.2	1.9	0.2	0.7†	0.1	36.4*	10.6
5	*Chaetoceros simplex*	1085/3	0.06†	0.03	0.75	0.4	1.1	0.2	0.2	0.1	19.6†	3.0
6	*Dunaliella polymorpha*	19/14	0.05	0.01	0.74	0.1	2.3	0.1	0.2	0.0	6.9	0.5
7	*Dunaliella quartolecta*	19/9	0.05	0.01	0.77	0.1	2.3	0.1	0.2	0.0	7.9	0.5
8	*Dunaliella salina*	19/30	0.05	0.01	0.82	0.1	2.5	0.2	0.2	0.0	8.9	0.4
9	*Dunaliella tertiolecta*	19/6B	0.05	0.01	0.76	0.1	2.3	0.1	0.2	0.0	8.8	0.6
10	*Tetraselmis apiculata*	66/15	0.05	0.01	1.03	0.1	1.0	0.1	0.2	0.0	18.0†	3.4
11	*Pleurochrysis dentata*	904/1	0.05	0.01	0.78	0.2	1.7	0.0	0.3	0.1	14.4	3.4
12	*Dunaliella polymorpha*	19/7A	0.04	0.00	0.71	0.0	2.2	0.1	0.2	0.0	8.1	0.3
13	*Pavlova salina*	940/3	0.04	0.01	0.56	0.1	1.3	0.1	0.3	0.0	20.0†	4.2
14	*Dunaliella bioculata*	19/4	0.04	0.00	0.64	0.1	2.1	0.1	0.2	0.0	7.3	0.7
15	*Dunaliella tertiolecta*	19/22	0.04	0.00	0.69	0.1	1.8	0.2	0.1	0.0	7.5	0.1

*Significantly different ($P<0.05$) from rest of the column except where denoted: †. All data for the first 4 parameters are above the 70th percentile for the screen. All strains were grown on f/2 unless denoted ‡JM. Full data are found in Supplementary Dataset S7 online.

Table S2 Strains producing high levels of total omega-3 long-chain PUFA

No.	Species	Strain CCAP No.	ω-3 PUFA Productivity (mgl⁻¹d⁻¹)	SD	ω-3 PUFA Yield (mgl⁻¹)	SD	ω-3 PUFA composition %Area	SD	ω-3 PUFA content %DW	SD	TFA content %DW	SD
1	*Chlorella vulgaris*	211/21A	1.90*	0.15	41.8*	3.4	17.9‡	0.2	9.2†	0.6	51.8*	4.2
2	*Chroomonas placoidea*	978/8	1.19	0.22	16.6	3.0	44.5	1.2	4.9	0.6	11.0	1.5
3	*Tisochrysis lutea*	927/14	1.15	0.06	16.1	0.8	43.1	1.0	4.9	0.4	11.4	0.6
4	*Dictyo. ehrenbergianum*§	222/1C	1.00	0.11	23.0	2.5	45.7	5.6	5.0	0.5	11.1	2.3
5	*Pleuro. pseudoroscoffensis*	913/2	0.82	0.12	13.1	1.9	37.7	0.4	4.2	0.4	11.2	1.2
6	*Prymnesium parvum*	946/4	0.81	0.17	17.9	3.7	28.8	0.2	4.7	0.6	16.1	2.1
7	*Pleurochrysis carterae*	961/4	0.74	0.03	11.9	0.4	36.4	1.4	4.7	0.3	12.9	1.3
8	*Pycnococcus provasolii*	190/1	0.74	0.11	11.9	1.7	34.6	1.7	5.2	0.7	15.0	1.9
9	*Dictyo. tetrachotomum*§	222/4	0.72	0.28	14.4	5.6	40.1	3.3	5.9	0.3	14.7	0.9
10	*Prymnesium parvum*	946/6	0.71	0.30	13.6	6.3	32.6	1.6	8.6	2.5	26.7	8.5
11	*Desmodesmus elegans*¶	258/8	0.65	0.29	24.6†	11.0	33.8	1.7	6.3†	1.9	18.9	6.2
12	*Isochrysis galbana*	927/1	0.58	0.08	11.6	1.5	34.4	0.3	5.5	0.4	16.0	1.1
13	*Amphidinium carterae*	1102/3	0.23‡	0.06	4.6‡	1.2	67.5*	0.7	9.3*	1.5	13.8	2.1

*Significantly different (P<0.05) from rest of the column except where denoted: †. All data are above the 70th percentile for the screen for the first 4 parameters except where indicated: ‡. All strains were grown on f/2 unless denoted §JM or ¶3NBBM+V. Full data set in Supplementary Dataset S7 online.

SUPPLEMENTARY TEXT

Text S1: FA composition

Detailed discussion of the FA compositional data in relation to phylogenetic origin is presented here along with a comparison of similar data in the literature. Strain disambiguation can account for many apparent outliers in the cluster analysis of FA composition shown in Fig. 5 and is discussed further. The data for the cluster analysis is also tabulated and depicted graphically (Supplementary Dataset S6 and Fig. S9 online). A phylogenetic tree is displayed for CCAP strains, or duplicate strains held in other protistan collections, included in the compositional analysis (Fig. 2). The cluster analysis of the FA data separated the green algae from the chromistan and red algae (Fig. 5). In particular, the distinct patterns of C_{16} desaturation have been attributed to different plastidial desaturase substrate specificities in the red and green algal lineages[1]. Clustering of FA compositional data led to further grouping of most strains by phyla, class and in some cases according to genus.

Among the green algae, C_{16} PUFA in double-bond position series n-3, 6, 9, 12 were generally present at minor levels and were diagnostic (Supplementary Dataset S6 and Fig. S9 online). High levels of 16:0, 18:1n-9, 18:2n-6 and 18:3n-3 also characterized green algae and C_{20} PUFAs were also present in most genera. In particular, *Tetraselmis* (Chlorodendrophyceae) species had appreciable EPA levels (1-6%) with Arachidonic acid (ARA or 20:4n-6), Eicosatetraenoic acid (ETA or 20:4n-3) and Stearidonic acid (SDA or 18:4n-3) (<6%) also present, as noted in earlier work[2–5]. This genus was distinguished from the other green algae by relatively high 18:1n-7 with above-trace levels of 17:0 (1-2%); a minor proportion of these FA might be attributable to marine bacterial consortia[2]. The Prasinophycean species (*Pyramimonas spinifera* CCAP 67/18 and *Pycnococcus provasoli* CCAP 190/1) were unusual among green alga in having appreciable levels of the C_{22} PUFA DHA: 5% in the latter species

and notably 18:4(n-3) was also high at 11%. Similar findings were noted with the synonymous *Pyc. provasoli* CS-185 and a *Pyr. cordata* strain[4]. The divergent FA composition of the Prasinophycean species from that of the other green algae was apparent in the cluster analysis (Fig. 5). It was of interest that *Pyc. spinifera* CCAP 67/18 was also divergent at the 18S level from the other green algal classes (Fig. 2).

Of the Chlorophyceaens, the *Dunaliella* investigated here had a degree of compositional variation with little divergence at the 18S level in the genus (Fig. 2). For instance, the minor FA 20:1n-9 reached 16% in *D. tertiolecta* CCAP 19/6B; not noted elsewhere in *Dunaliella* (Supplementary Dataset S6 and Fig. S9 online)[2,3,6–8]. In *D. primolecta* CCAP 11/34 and *D. maritima* CCAP 19/1, a shift towards saturation (high 18:0 and 18:1n-9) might be attributed to higher TFA levels at 32% and 14% compared with the rest of the genus examined, which primarily accumulated carbohydrate (Supplementary Dataset S4 and 6 online, Table 3)[9]. Among the other Chlorophyceaens, *Haematococcos pluvialis* strains examined here (CCAP 34/6 and CCAP 34/1F) were distinguished by high 18:2n-6 (28%) and presence of ARA and EPA as noted for other *H. pluvialis* strains elsewhere[7,10]. *D. elegans* CCAP 258/8 was distinguished by relatively high SDA (5%), as noted in this genus in previous work[7,11], but unique to this strain or its analysis, there was appreciable 14:0 (3%) (Supplementary Dataset S6 and Fig. S9 online).

A diverse group of Trebouxiophyceae were examined, including marine and fresh-water green coccoid algae. This included 5 morphologically '*Chlorella*-like' algae isolated from marine environments (termed 'marine *Chlorella*' and comprising *Chlorella* and *Chloroidium*) (Supplementary Dataset S1 online). FA compositions were very similar between *C. vulgaris* CCAP 211/21A and CCAP 211/75 strains; consistent with the 18S based phylogeny which indicated that they are closely related (Fig. 2 and 5). Both of these strains have recently been re-assigned as marine forms of *C. vulgaris* (T. Proeschold pers. comm.). Although the former strain was found to accumulate higher TFA levels: 52% c.f. 30% DW (Table 2, Supplementary

Dataset S4 online). Formerly known as *C. marina*, *Chloroidium saccharophilum* CCAP 211/27 FA composition was divergent from the *C. vulgaris* strains, perhaps reflecting its evolutionary divergence from the *Chlorella* (Fig. 2 and 5). *C. spaerkii* CCAP 211/29A and *C. stigmatophora* CCAP 211/20 differed from *C. vulgaris* strains examined here in having detectable C_{20} and C_{22} PUFA (principally EPA, ETA: 20:4n-3, and traces of DHA and 22:4n-6). In having appreciable 20:1n-9, 16:4n-3 and SDA, the *Chlorella* sp. CCAP 211/53 strain resembled more that of the *Dunaliella* in terms of FA composition as apparent in the cluster analysis (Fig. 5). This CCAP 211/53 strain was also phylogenetically more related to the *Dunaliella* than *Chlorella* (Fig. 2) but does not resemble this genus in terms of morphology (www.ccap.ac.uk).

Similarity was evident between the *Stichococcus bacillaris* CCAP 379/5 and 35 strains, with *Pseudostichococcus monoallantoides* CCAP 364/1, both in terms of FA profile and phylogenetic data (Fig. 2 and 5; Supplementary Fig. S9 online). These marine strains were distinguished among Trebouxiophyceans by the presence of 3-4 mol% C_{20} PUFA principally as ARA, as noted in some strains in the SAG collection[7], but higher 14:0 levels were noted in our study (<3 mol%). This group also resembled the freshwater *Dictyosphaerium*, *Mucidospherium* and *Heynigia* strains in having appreciable 14:0 (Supplementary Dataset S6 and Fig. S9 online). The latter 3 genera were formerly grouped in *Dictyosphaerium* and their FA profiles mostly clustered together: in fact *M. pulchellum* (CCAP 222/2A and 222/2B), *D. tetrachotum* CCAP 222/4, *D. sphagnale* CCAP 222/13 and *H. dictyospheroides* CCAP 222/2D were all similar in this respect (Fig. 5). Variation occurred in the minor FA 16:4n-3, SDA and particularly in GLA, which was notably high in the outlier *D. ehrenbergianum* CCAP 222/1A (6%), but absent in an alternate strain of this taxon: CCAP 222/1C (Supplementary Dataset S6 and Fig. S9 online). The presence of 14:0 and high GLA has not previously been noted in *Dictyosphaerium*[7].

Within the chromistan and red algal lineages, C_{16} PUFA series n-1, 4, 7, 10 prevailed, with at least 16:1n-7 being present (Supplementary Dataset S6 and Fig. S9 online). The

chromistan plastid is thought to have evolved from endocytobiotic engulfing of an ancestral red alga and the distinctive C_{16} desaturation patterns in the red and green algal lineages appear to reflect this[1]. The red algal classes Rhodophyceae and Porphyridiaceae were distinguished by high levels of both ARA (20-30%) and EPA. A similar compositional profile was observed with the Glaucophycean *Cyanophora paradoxa* CCAP 981/1 and this was also evident in some *C. paradoxa* SAG collection strains (Fig. 5, Supplementary Dataset S6 and Fig. S9 online)[7]. Despite this similarity, there was considerable evolutionary distance between these three classes (Fig. 2).

Haptophyte FA composition closely followed taxonomic boundaries (Fig. 2 and 5; Supplementary Dataset S6 and Fig. S9 online). Coccoliths were distinguished from Palovophyceans *(Diacronema*, *Pavlova*) by a preponderance of C_{22} PUFA (DHA and/or Docasapentaenoic acid: DPA, mostly as the 22:5n-6 isoform rather than 22:5n-3) over C_{20} PUFA (EPA) and by the presence of appreciable 18:5n-3, which was completely absent in the latter class. Palovophyceans on the other hand were characterized by equal amounts of C_{22} and C_{20} PUFA and unlike Coccoliths, substantial 16:1n-7. This was in agreement with earlier datasets from mostly different sp./strains of *Chrysotila*, *Isochrysis* and *Pavlova*[3,5,6,12]. Among the Coccolithophyceaen orders, Isochrysidales (*Chrysotila* and *Isochrysis*) and Prymnesiales (*Prymnesium*) differed somewhat from Coccolithales (*Pleurochrysis*). The latter stood out among the haptophytes examined in lacking substantial levels of DPA (22:5n-6) or 14:0, whereas 14:0 was higher (12-28%) in the former orders. Considerable levels of 14:0 also being a characteristic of the Pavlovophyceans[12].

According to the FA cluster analysis, *Pavlova gyrans* CCAP 940/1B, *P. pinguis* CCAP 940/2 and *P. salina* CCAP 940/3 clustered in a separate group from *Diacronema lutheri* CCAP 931/7 and *Diacronema vilkianum* CCAP 914/1 (Fig. 5). The former were characterized by the

unusual FA 22:4n-6 (Docosatetraenoic acid or adrenic acid) at 1% levels. The separation was supported by the 18S rDNA phylogenetic data which in turn was in agreement with previous findings (Fig. 2)[13]. The FA cluster analysis also separated the Pavlovophyceans from Coccoliths, where the following genera *Isochrysis*, *Tisochrysis*, *Chrysotila*, *Pleurochrysis* and *Prymnesium* were grouped together. The species belonging to these genera also clustered by genus according to FA composition, in good agreement with the 18S data (Fig. 2 and 5).

Two strains described as Dictyochophyceaen: *Pedinella marina* CCAP 941/1A and *Pedinella* sp. 941/3 are probable haptophytes (I. Probert pers. comm.) and cluster with the Isochrysidales by FA composition (Fig. 5). *P. marina* CCAP 941/1A differed from other haptophytes examined here in having moderate levels (2%) of the scarce C_{20} PUFA 20:2n-6 (Eicosadienoic acid). A *Chromulina ochromonoides* CCAP 909/1 strain (Chrysophyceae) also closely resembled the Isochrysidales in terms of FA composition (Supplementary Dataset S6 and Fig. S9 online). The lipid composition of two cryptophytes; *Chroomonas placoidae* CCAP 978/8 and *Rhodomonas reticulata* CCAP 995/2 were similar to each other and resembled haptophytes in having prominent 14:0, 18:1n-9, 18:2n-6, 18:3n-3, SDA and EPA. However, differences in their C_{20-22} PUFA profile were noted, with the former Cryptophycean distinguished by minor levels of DPA (22:5n-6) and the relatively rare ETA (20:4n-3) in place of ARA and DHA in the latter. These observations were in agreement with an earlier study on various *Rhodomonas* sp. and *C. placoidae* CS-200, which is synonymous with the CCAP 978/8 strain[14]. In a further study, another *Chroomonas* species, *C. salina* CS-174 did not differ from the above *Rhodomonas* in this regard[3]. The dinoflagellate *Amphidinium carterae* CCAP 1102/3 was characterized by particularly high combined levels of omega-3 long-chain PUFA's: SDA (35%), DHA (18%) and EPA (15%) (Supplementary Dataset S6 and Fig. S9 online). Similar levels were noted in a UTEX 1687 strain, which was particularly high in SDA, but this profile was not

observed in the SAG collection strains of this species[7,15]. It further resembled many haptophytes in having very minor long-chain C_{18-24} saturates and trace 18:5n-3[16].

Heterokont algae of the class Eustigmatophyceae and diatoms (Bacillariophyceae) were characterized by high 16:1n-7, in conjunction with approximately equal amounts of 16:0 (or 14:0) and minor to substantial EPA levels (Supplementary Dataset S6 and Fig. S9 online). These were the principal FA among the Eustigmatophycean genera with *Nannochloropsis*, as noted by previous studies[17,18]. FA composition was particularly uniform here, with 16:0 and 16:1n-7 being the dominant FA with appreciable 14:0, giving rise to low polyunsaturation levels and short average chain lengths. The C_{20} PUFA's were notably higher in the genera *Monodopsis* and *Eustigmatos*; with 22 mol% EPA and 4% ARA (Supplementary Dataset S6 and Fig. S9 online), as noted in the literature for *M. subterranea* CCAP 848/1[19].

Diatom FA profiles were complex and were distinguished from other Heterokont algae screened here by having more substantial levels of C_{16} n-1,4,7,10 series PUFA (Supplementary Dataset S6 and Fig. S9 online). These comprised mainly 16:2n-4 (6% in *Chaetoceros curvisetus* CCAP 1010/12; *Navicula pellicosa* CCAP 1050/9) and 16:3n-4 (12% in *Attheya* sp. CCAP 1010/15), with 16:4n-1 (6% in *Odontella mobiliensis* CCAP 1054/4; *Porosira pseudodenticulata* CCAP 1060/4) and 16:2n-7 often present; these findings were in agreement with earlier data from several other diatom species[3,20]. This was consistent with flux through plastidial Δ9 desaturation of 16:0 to 16:1n-7 with further desaturation in the plastid (Δ6, Δ12, Δ15) as proposed for *Phaedactylum*[1]. Within the data-set generated in this study, 16:3n-4 was virtually diagnostic for diatoms, whereas 16:2n-4 was also seen in haptophytes at low levels. Of the C_{18} FA, 18:1n-7 was often found to exceed 18:1n-9 in diatoms and was occasionally abundant, e.g. *Thalassiosira rotula* CCAP 1085/20 and CCAP 1085/22 (11% and 36%). High levels of 18:1n-7 have been noted in other diatom sp./strains in the literature at up to 5%[2,3,20]. In all these cases,

elongation of 16:1n-7 to 18:1n-7 might be occurring at the expense of further plastidial desaturation; indeed C_{16} PUFA's were much reduced in the *T. rotula* CCAP 1085/22 strain, where 18:1n-7 was most abundant. Minor amounts of n-4, 7, 10 series C_{18} PUFA FA were frequently present as 18:3n-4 and 18:4n-4, e.g. *Navicula pelliculosa* CCAP 1050/9 at 1-2%, again suggesting elongation of unsaturated C_{16}. Slightly greater amounts of n-3, 6, 9, 12 series C_{18} PUFA FA were also present in the diatoms. For instance, SDA (18:4n-3) at 4-8% in the *Porosira*, and some *Thalassiosira* and *Chaetoceros* species. Minor levels of GLA (18:3n-6) were present at 2-3% (*Cylindrotheca* sp. CCAP 1017/7; *Ch. muelleri* CCAP 1010/3); 18:3n-3 at 3% (*Stauroneis simulans* 1078/1) and 18:2n-6 also at 3% (*Cylindrotheca* sp. CCAP 1017/7). C_{20} PUFA's were usually in the form of EPA which ranged from 4-30%, with the highest content in the polar strain *Attheya* sp. CCAP 1010/15. In addition, ARA, in association with comparable levels of EPA, was noted in *Cylindrotheca* species (10%) and *Ch. muelleri* CCAP 1010/3 (5%). Overall, similar maximum values of EPA and ARA were reported in other diatoms elsewhere in the literature, e.g. respectively *Amphora* sp. CS-10 and *Fragilaria* sp. GOC1[20,21]. DHA was generally present at minor levels in all diatoms (0.3-5%) and was the principal C_{22} PUFA; a range also noted elsewhere in other diatoms[3,20,21]. Of the saturates, 14:0 levels varied widely from very low in A*chnanthes,* to 30-40% (*Odontella mobiliensis* CCAP 1054/4; *Cylindrotheca sp.* CCAP 1017/7; *Ch. debilis* CCAP 1010/6); with levels of up to 30% noted in the literature in other diatom species and strains[3,20,21].

Although certain FA's were characteristic of the diatoms, much within-genus variation was evident (e.g. for the *Odontella*, *Navicula*, *Chaetoceros* and *Thalassiosira* genera); even between strains of the same species (e.g. *Th. rotula*). Additionally, evolutionary divergence between species/strains was also marked in these examples on the basis of molecular barcode data (Fig. 2 and 5). Perhaps as a consequence, there was no clear distinction between the diatom classes Bacillariophyceae and Coscinodiscophyceae at the FA level in the cluster

analysis (Fig. 5), although this division was evident on the basis of the 18S rDNA data (Fig. 2). Nevertheless, the *Nitzschia*, *Achnanthes* and *Porosira* species and strains did show within-genus similarities in FA composition, although the species of the former genus were evolutionary divergent on the basis of molecular data (Fig. 2 and 5).

SUPPLEMENTARY REFERENCES

1. Domergue, F. *et al.* New Insight into *Phaeodactylum tricornutum* Fatty Acid Metabolism. Cloning and Functional Characterization of Plastidial and Microsomal 12-Fatty Acid Desaturases. *Plant Physiol.* **131,** 1648–1660 (2003).

2. Viso, A.-C. & Marty, J.-C. Fatty acids from 28 marine microalgae. *Phytochemistry* **34,** 1521–1533 (1993).

3. Volkman, J. K., Jeffrey, S. W., Nichols, P. D., Rogers, G. I. & Garland, C. D. Fatty acid and lipid composition of 10 species of microalgae used in mariculture. *J. Exp. Mar. Bio. Ecol.* **128,** 219–240 (1989).

4. Dunstan, G. A., Volkman, J. K., Jeffrey, S. W. & Barrett, S. M. Biochemical composition of microalgae from the green algal classes Chlorophyceae and Prasinophyceae. 2. Lipid classes and fatty acids. *J. Exp. Mar. Bio. Ecol.* **161,** 115–134 (1992).

5. Renaud, S. M., Thinh, L.-V. & Parry, D. L. The gross chemical composition and fatty acid composition of 18 species of tropical Australian microalgae for possible use in mariculture. *Aquaculture* **170,** 147–159 (1999).

6. Zhukova, N. V & Aizdaicher, N. A. Fatty acid composition of 15 species of marine microalgae. *Phytochemistry* **39,** 351–356 (1995).

7. Lang, I., Hodac, L., Friedl, T. & Feussner, I. Fatty acid profiles and their distribution patterns in microalgae: a comprehensive analysis of more than 2000 strains from the SAG culture collection. *BMC Plant Biol.* **11,** 124 (2011).

8. Mooney, H. M., Cooney, J. J., Baisden, C. M. & Patching, J. W. Fatty acids of *Dunaliella tertiolecta* and *Skeletonema costatum* Grown in the Presence of Phenyltin Compounds. *Bot. Mar.* **38,** 423–429 (1995).

9. Roessler, P. G. Environment control of glycerolipid metabolism in microalgae: commercial implications and future research directions. *J. Phycol.* **26,** 393–399 (1990).

10. Zhekisheva, M., Boussiba, S., Khozin-goldberg, I., Zarka, A. & Cohen, Z. Accumulation of oleic acid in *Haematococcus pluvialis* (Chlorophyceae) under nitrogen starvation or high light is correlated with that of Astaxanthin esters. *J. Phycol.* **331,** 325–331 (2002).

11. Kaur, S., Sarkar, M., Srivastava, R. B., Gogoi, H. K. & Kalita, M. C. Fatty acid profiling and molecular characterization of some freshwater microalgae from India with potential for biodiesel production. *N. Biotechnol.* **29,** 332–344 (2012).

12. Volkman, J. K., Dunstan, G. A., Jeffrey, S. W. & Kearney, P. S. Fatty acids from microalgae of the genus Pavlova. *Phytochemistry* **30,** 1855–1859 (1991).

13. Bendif, E. M. *et al.* Integrative taxonomy of the Pavlovophyceae (Haptophyta): a reassessment. *Protist* **162,** 738–761 (2011).

14. Dunstan, G. A., Brown, M. R. & Volkman, J. K. Cryptophyceae and rhodophyceae; chemotaxonomy, phylogeny, and application. *Phytochemistry* **66,** 2557–2570 (2005).

15. Leblond, J. D. & Chapman, P. J. Lipid class distribution of highly unsaturated long chain fatty acids in marine dinoflagelates. *J. Phycol.* **36,** 1103–1108 (2000).

16. Okuyama, H., Kogame, K. & Takeda, S. Phyogenetic significance of the limited distribution of octadecapentaenoic acid in Prymnesiophytes and photosynthetic Dinoflagellates. *Proc. NIPR Symp. Polar Biol.* **6,** 21–26 (1993).

17. Volkman, J. K., Brown, M. R., Dunstan, G. A. & Jeffrey, S. W. The biochemical composition of marine microalgae from the class Eustigmatophyceae. *J. Phycol.* **29,** 69–78 (1993).

18. Ben-Amotz, A., Tornabene, T. G. & Thomas, W. H. Chemical profile of selected species of microalgae with emphasis on lipids. *J. Phycol.* **21,** 72–81 (1985).

19. Cohen, Z. Production potential of eicosapentaenoic acid by *Monodus subterraneus*. *J. Am. Oil Chem. Soc.* **71,** 941–945 (1994).

20. Dunstan, G. A., Volkman, J. K., Barrett, S. M., Leroi, J.-M. & Jeffrey, S. W. Essential polyunsaturated fatty acids from 14 species of diatom (Bacillariophyceae). *Phytochemistry* **35,** 155–161 (1993).

21. Renaud, S. M., Thinh, L.-V., Lambrinidis, G. & Parry, D. L. Effect of temperature on growth, chemical composition and fatty acid composition of tropical Australian microalgae grown in batch cultures. *Aquaculture* **211,** 195–214 (2002).
